# Relative validity of food and nutrient intakes derived from a brief-type diet history questionnaire for Japanese children and adolescents

**DOI:** 10.1017/S0007114525104042

**Published:** 2025-09-14

**Authors:** Hitomi Okubo, Ryoko Tajima, Nana Shinozaki, Shizuko Masayasu, Satoshi Sasaki, Kentaro Murakami

**Affiliations:** 1 Department of Nutritional Epidemiology and Behavioural Nutrition, Graduate School of Medicine, The University of Tokyo, 7-3-1 Hongo, Bunkyo-ku, Tokyo 113-0033, Japan; 2 Department of Social and Preventive Epidemiology, School of Public Health, The University of Tokyo, 7-3-1 Hongo, Bunkyo-ku, Tokyo 113-0033, Japan; 3 Ikurien-Naka, 3799-6 Sugaya, Naka-shi, Ibaraki 311-0105, Japan

**Keywords:** Diet history questionnaire, Relative validity, Nutrient intake, Food group intake, Children, Adolescents

## Abstract

The aim of this study was to evaluate the relative validity of food and nutrient intakes estimated by a brief-type diet history questionnaire for Japanese children and adolescents (BDHQ15y) designed to assess habitual dietary intake during the previous month. A total of 432 boys and 412 girls aged 6–17 years from thirty-two prefectures in Japan completed the BDHQ15y and subsequently provided 8-day weighed dietary records (DR) on two non-consecutive days over four seasons for comparison. Among the intakes of forty-four nutrients and thirty-one food groups adjusted for energy intake using the density model, the BDHQ15y showed percentage differences in median intake of less than 10 % compared with the DR for nineteen nutrients in both sexes, as well as for eleven and seven food groups in boys and girls, respectively, indicating good agreement for key nutrients and food groups, including protein, fat, carbohydrate, dietary fibre, grains, vegetables, dairy products and sugar-sweetened beverages (SSB). The median values (25th–75th percentiles) of Spearman’s correlation coefficients in boys and girls were 0·33 (0·28–0·38) and 0·28 (0·23–0·35) for nutrients, respectively, and 0·36 (0·29–0·42) and 0·29 (0·24–0·36) for food groups, respectively. Bland–Altman plots showed wide limits of agreement, with overestimation at higher intakes for most nutrients and food groups, except SSB. In conclusion, the BDHQ15y shows promise for large-scale dietary monitoring, particularly for estimating group-level intakes of key nutrients and food groups. However, its limited ability to rank individual intakes and the variability in individual-level assessments necessitate cautious interpretation and application.

Accurate assessment of dietary intake is essential to understand how dietary intake influences health outcomes, particularly during childhood and adolescence, which are periods of rapid physical growth and cognitive development^(1)^. Dietary habits formed during these stages often persist into adulthood, shaping long-term health trajectories and affecting both immediate well-being and the risk of chronic diseases in later life^(2,3)^; therefore, valid dietary assessment tools are crucial to inform public health strategies and interventions.

Dietary questionnaires, such as food frequency questionnaire (FFQ) and diet history questionnaires (DHQ), are commonly used for dietary assessment due to their time efficiency, lower cost and reduced respondent and researcher burden compared with traditional methods like dietary records (DR) and 24-h recalls^(4–6)^. In large-scale epidemiological studies, dietary questionnaires provide a feasible approach to assess long-term habitual diets and their relationships with health outcomes and disease risk^(4–6)^. However, assessing dietary intake in children and adolescents presents unique challenges^(7,8)^. As children transition into adolescence, they experience increased autonomy in food choices, are influenced by peer pressure and family dynamics and develop their personal preferences, leading to greater variability in their diets^(9,10)^. Additionally, cognitive development during this period can hinder the accuracy of dietary recall, as children’s limited memory capacity and developing sense of time make it difficult to report food consumption precisely^(11,12)^. These developmental limitations highlight the importance of validating dietary assessment tools to ensure reliable capture of habitual intake in this age group^(8,13)^.

To address these challenges, various dietary questionnaires have been adapted for children and adolescents^(14–24)^, including the brief-type diet history questionnaire for Japanese children and adolescents (BDHQ15y)^(22–24)^. This tool was developed based on the existing comprehensive and brief versions of a validated self-administered DHQ for Japanese adults^(25–29)^. Previous studies assessed the validity of the BDHQ15y using biomarkers of dietary intake as a reference, but these evaluations were limited to a specific demographic and geographic region in Japan, focusing on a narrow age range^(22–24)^. More importantly, the ability of the BDHQ15y to estimate a comprehensive range of nutrients and food groups remains underexplored. Given the widespread use of the BDHQ15y in large-scale epidemiological studies across Japan^(30,31)^, it is essential to extend its validation to a wider range of populations and age groups in order to ensure its relevance and accuracy for broader public health applications.

Therefore, the present study aimed to evaluate the relative validity of the BDHQ15y among Japanese children and adolescents aged 6–17 years in comparison with the 8-day weighed DR, with the goal of determining whether the BDHQ15y can accurately capture habitual dietary intake across a range of nutrients and food groups in this age group.

## Methods

### Study procedure and participants

This study used data from the Ministry of Health, Labour and Welfare-sponsored Nationwide Study on Dietary Intake Evaluation (MINNADE study), which aims to evaluate and develop methods for assessing the intake of hazardous environmental chemicals through foods in the Japanese population. Detailed descriptions on the study design and protocol have been previously published^(31–33)^. In brief, healthy, community-dwelling Japanese individuals aged 1–79 years from thirty-two of the forty-seven prefectures in Japan, covering over 85 % of the population, were recruited based on feasibility and regional population proportions. The research dietitians in each prefecture (*n* 453) used snowball sampling to recruit eligible, free-living individuals who could conduct a DR independently or with the help of a guardian for children. Exclusion criteria included dietitians and their cohabitants, individuals receiving dietary counselling from a medical doctor or dietitian, those undergoing insulin or dialysis treatment, pregnant or lactating women and infants exclusively consuming human milk. Only one participant per household was eligible.

Data were collected over three 1-year rounds: November 2016 to September 2017, October 2017 to September 2018 and November 2019 to August 2020. A total of 2304 participants were recruited in the first round, comprising 256 individuals (128 males and 128 females) from each of nine age bands (1–6, 7–13, 14–19, 20–29, 30–39, 40–49, 50–59, 60–69 and 70–79 years). To account for dropouts in the first round, 110–119 individuals per sex and age group were recruited in the second round, resulting in 2051 individuals invited to participate. Additionally, 438 children aged 1–6 years were recruited in the third round to gather data on younger children. Due to the snowball sampling method, the number of individuals contacted was not recorded; thus, the response rate could not be calculated. In total, 4736 individuals (first round: *n* 2263, second round: *n* 2036 and third round: *n* 437) agreed to participate in the MINNADE study. Participants were asked to first complete the BDHQ and then, after a designated interval, complete the DR on two non-consecutive days for each of the four seasons, totalling 8 days of records. The protocol for the MINNADE study was in accordance with the guidelines of the Declaration of Helsinki and was reviewed and approved by the Ethics Committee of the University of Tokyo Faculty of Medicine (protocol codes: 11397 and 2019124NI; approval dates: 25 October 2016 and 17 October 2019, respectively). Written informed consent was obtained from all participants, or from parents or guardians in the case of participants under 18 years of age.

**Figure 1.**
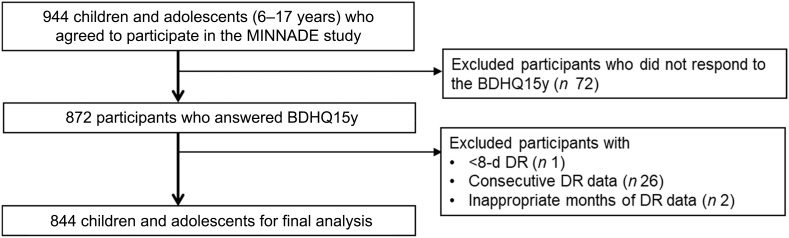
Flow diagram of study subject selection.

The present analysis focused on children and adolescents aged 6–17 years with at least 1-day data of DR (*n* 944) (Figure 1). Participants who did not complete the BDHQ15y (*n* 72), those with fewer than 8 d of DR data (*n* 1), those who conducted DR on consecutive days (*n* 26) and those whose DR were not conducted in the appropriate months (i.e. October, November and December for autumn; January, February and March for winter; April, May and June for spring and July, August and September for summer (*n* 2)) were excluded. Consequently, data from 844 children and adolescents aged 6–17 years were used in the present analysis.

### Brief-type diet history questionnaire for Japanese children and adolescents

The BDHQ15y is a four-page, structured questionnaire designed to assess dietary habits over the preceding month for Japanese children and adolescents. It was developed in 2005 based on comprehensive and brief versions of a validated self-administered DHQ for Japanese adults^(25–29)^. The BDHQ15y consists of the following four sections: (i) intake frequencies of sixty-one food and beverage items; (ii) daily intake of rice, including type of rice (refined and unrefined) and size of rice bowl; (iii) usual cooking methods for fish and meat dishes and (iv) general dietary behaviours (e.g. preferences for fatty cuts of meat and amount of soup consumed with noodles). The BDHQ15y also includes questions on basic characteristics of the respondent, such as sex, date of birth, self-reported height and weight, person who mainly completed the questionnaire and physical activity. Written instructions on how to complete the questionnaire are provided at the beginning, along with sample responses.

Most of the food and beverage items in the BDHQ15y were selected from the adult version of the brief type self-administered DHQ (BDHQ)^(28)^, with a focus on those commonly consumed in Japan. The BDHQ3y, validated for Japanese children aged 3–6 years^(34)^, was also referenced to help inform the selection of certain items, particularly for younger children in the target age range. For the BDHQ15y, several items, such as alcoholic beverages, were excluded, while others frequently consumed by children and adolescents, such as yoghurt, cheese, fried potatoes, chocolate, bread spreads, ketchup and nutritional snacks (e.g. CalorieMate and Weider in Jelly), were included based on general dietary trends and expert judgement to better capture dietary habits among Japanese youth. Seven predefined frequency scales, ranging from ‘did not drink/consume’ to ‘two times or more per day’ for food items or ‘four cups or more per day’ for beverage items, were used. In the BDHQ15y, information on portion sizes was not collected directly; instead, fixed portion sizes specific to sex and age were applied to all participants in the calculation of dietary intake. These standard portion sizes were derived from the adult version of the BDHQ, which was based on recipe books that generally represent the portion size for middle-aged women around 50 years old, who are primarily responsible for meal preparation^(28,29)^. Portion sizes for children and adolescents were determined by adjusting the reference value of the estimated energy requirement for 50-year-old women using age- and sex-specific estimated energy requirement ratios^(35)^.

Dietary intakes of three food items commonly added during cooking, namely, salt, fat and oil and sugar, were estimated according to the diet history method, using the qualitative information from sections 3 and 4. Intakes of table salt and salt-containing seasonings at the table, such as soya sauce and soup consumed with noodles, were estimated from answers to the corresponding qualitative questions in section 4. Additionally, individual preferences for meat fat content were incorporated to refine the nutritional composition values, ensuring that the fat content was consistent with the selection of lean or fatty meat. Estimates of daily intakes of foods (68 food items in total; see online Supplementary Table ST1), energy and selected nutrients were calculated using an *ad hoc* computer algorithm for the BDHQ15y, which was based on the BDHQ^(28,29)^. The nutritional values (i.e. energy and nutrient contents) of each food group item were established using the 2010 version of the Standard Tables of Food Composition in Japan^(36)^, partly supported by the 2015 version^(37)^. For food items without data on the added sugar content, added sugar values were derived from the same or similar food items in the 2011–2012 Food Patterns Equivalents Database^(38)^. Teaspoon equivalents in the Food Patterns Equivalents Database were converted into grams by multiplying by 4·2 (grams of added sugar per teaspoon).

### Eight-day weighed dietary record

Information on actual dietary intake was collected using two-non-consecutive-day weighed DR for each of the four seasons (8 d in total). In this study, the 8-day DR was used as the reference method. Details on the procedure of DR have been described elsewhere^(31–33)^. Briefly, each participant recorded two days of dietary intake per season: half of participants recorded two weekdays (Monday to Friday), while the other half recorded one weekday and one weekend day (Saturday, Sunday or national holidays). This design aimed to capture an approximate representation of overall dietary habits with a 3:1 ratio of weekdays to weekend days (actual ratio 5:2), while maintaining survey feasibility. After receiving verbal and written instructions by a research dietitian, as well as an example of a completed diary sheet, each participant was requested to weigh and record all foods and beverages consumed, both inside and outside the home, using a digital scale (KS-812WT; Tanita, Tokyo, Japan), which measures up to 2 kg in 1 g increments. Parental or guardian assistance was encouraged for younger participants as needed. To ensure the accuracy of reported information on foods consumed outside the home – such as school lunches and snacks – parents were instructed, in accordance with the dietary assessment manual, to ask their children about the types and portion sizes of foods consumed and to supplement this information using external sources (e.g. school lunch menus or newsletters).

Within a few days after each recording day (usually the next day), the research dietitian collected the recording diary, checked the completeness of recording and recorded additional information if necessary. All the collected diaries were checked by trained dietitians at the central office in terms of coding, recorded weights and descriptions of items consumed. Finally, estimates of daily intakes of energy and nutrients were calculated using the 2015 version of the Standard Tables of Food Composition in Japan^(37)^. Dietary supplements were not considered during calculation of nutrient intake because a nutrient composition database of dietary supplements in Japan is lacking.

### Statistical analyses

All statistical analyses were conducted separately for boys and girls using SAS statistical software (version 9.4, SAS Institute, Inc.). The dietary variables analysed in this study comprised energy, forty-four nutrients and thirty-one food groups (see online Supplementary Table ST1). Descriptive statistics for participant characteristics were presented as means and SD, while dietary data were summarised as medians with 25th and 75th percentiles to account for their skewed distributions. Energy adjustment was primarily conducted using the density model for both nutrient and food group intakes; however, for nutrients, the residual model^(4)^, which is widely recognised and commonly used in epidemiological studies, was also applied.

To evaluate estimation accuracy at the group level, percentage differences in median intake between the two methods (i.e. the BDHQ15y and 8-day DR) were calculated using the following formula: (BDHQ15y – 8-day DR)/8-day DR × 100. Percentage differences in median intakes were considered good (≤ 10·0 %), acceptable (10·1–20·0 %) and poor (> 20·0 %) with reference to thresholds suggested in prior research^(39)^. The ability of the BDHQ15y to rank individuals within the population was assessed by calculating Spearman’s rank correlation coefficients for energy-adjusted intakes of nutrients and food groups derived from both the BDHQ15y and 8-day DR. Agreement between the two methods was further evaluated using Bland–Altman plots^(40)^ for selected variables, including protein, fat, carbohydrate, total vegetables, fruit, dairy and sugar-sweetened beverages (SSB) because these are key macronutrients and food groups with direct implications for dietary intake and health outcomes and are particularly relevant to public health nutrition for this age group^(41)^. Proportional bias between the two methods was tested using linear regression analysis, with statistical significance set at *P* < 0·05. As a sensitivity analysis, evaluations were stratified by sex and age groups (6–9, 10–14 and 15–17 years), with results detailed in the online Supplementary Materials.

## Results

This study included 432 boys and 412 girls (Table 1). The mean ages of boys and girls were 11·8 years (sd 3·4 years) and 11·5 years (sd 3·5 years), respectively.

**Table 1.** Basic characteristics of study subjects (Mean values and standard deviations)

	Boys								Girls							
	All (*n* 432)		Age groups						All (*n* 412)		Age groups					
			6–9 years (*n* 126)		10–14 years (*n* 184)		15–17 years (*n* 122)				6–9 years (*n* 144)		10–14 years (*n* 162)		15–17 years (*n* 106)	
	Mean	sd	Mean	sd	Mean	sd	Mean	sd	Mean	sd	Mean	sd	Mean	sd	Mean	sd
Age (years)	11·8	3·4	7·6	1·1	12·1	1·5	15·9	0·8	11·5	3·5	7·5	1·1	12·0	1·6	16·0	0·8
Body height (cm)	149·3	19·7	125·6	9·2	152·1	12·6	169·8	5·5	142·9	15·7	124·9	8·9	149·2	8·2	157·5	5·8
Body weight (kg)	43·2	16·0	26·4	6·5	43·8	11·9	59·6	9·2	38·3	12·1	25·9	6·8	41·2	7·4	50·7	6·8
BMI (kg/m^2^)^*^	18·6	3·1	16·5	2·3	18·6	2·9	20·7	2·8	18·2	2·9	16·4	2·4	18·4	2·2	20·4	2·5

* BMI was calculated by dividing body weight (kg) by the square of height (m), based on self-reported values.

The median values of daily energy and energy-adjusted nutrient intakes derived from the 8-day DR and BDHQ15y are presented in Table 2 (for boys) and Table 3 (for girls). The median energy intakes estimated from the BDHQ15y closely aligned with those estimated from the 8-day DR, with percentage differences within 4 % for both sexes. Among forty-four nutrients examined, 19 (43 %) showed differences within 10 % between the BDHQ15y and 8-day DR for both sexes and both energy-adjusted models, except for *β*-carotene in the residual model for girls. These nutrients included protein, total fat, SFA, PUFA, cholesterol, carbohydrate, dietary fibre, *β*-carotene equivalent, *α*-tocopherol, niacin, vitamin B_6_, Na, potassium, Mg, phosphorus and Zn, indicating good agreement at the group level. In contrast, the BDHQ15y overestimated fifteen nutrients in boys and fourteen nutrients in girls by more than 20 % and underestimated two nutrients (*α*-carotene and thiamine) by more than 20 % in both sexes, indicating poor accuracy. This trend was consistent across both energy-adjusted models.

**Table 2.** Median estimates of daily energy and energy-adjusted nutrient intakes using the density and residual methods from the 8-day weighed dietary records (DR) and the brief-type diet history questionnaire for Japanese adolescents and children (BDHQ15y) in 432 Japanese boys aged 6–17 years: percentage differences in median intakes and Spearman’s rank correlations coefficients (CC)

	Energy-adjusted by the density method										Energy-adjusted by the residual method							
	Unit	8-day DR			BDHQ15y			Median difference (%)^§^	Spearman’s rank CC	Unit	8-day DR			BDHQ15y			Median difference (%)^§^	Spear man’s rank CC
		Median	P25	P75	Median	P25	P75				Median	P25	P75	Median	P25	P75		
Energy	–	–		–	–		–		–	kcal/d	2332	1917	2923	2247	1812	2872	–3·7	0·67
Protein	% energy	14·1	13·1	15·0	13·6	12·4	15·2	–3·7	0·33	g/d	77·7	72·3	83·0	73·8	67·7	82·2	–5·0	0·30
Fat	% energy	30·9	28·2	33·2	29·0	25·3	32·3	–6·1	0·31	g/d	75·4	69·3	81·0	69·8	61·4	78·7	–7·4	0·30
SFA	% energy	9·8	8·9	11·0	9·1	7·8	10·7	–7·3	0·37	g/d	24·1	21·6	27·2	22·2	18·9	26·1	–8·0	0·35
MUFA	% energy	11·6	10·5	12·7	10·0	8·5	11·1	–13·9	0·26	g/d	28·3	25·6	31·0	23·9	20·7	27·1	–15·6	0·27
PUFA	% energy	5·6	5·0	6·2	6·1	5·3	6·8	8·8	0·29	g/d	13·7	12·3	15·2	14·7	12·9	16·7	7·7	0·27
*n*-6 PUFA	% energy	4·3	3·9	4·9	5·1	4·5	5·8	18·2	0·23	g/d	10·6	9·5	11·8	12·5	10·8	14·1	17·8	0·21
*n*-3 PUFA	% energy	0·82	0·68	0·99	1·06	0·89	1·23	29·3	0·30	g/d	2·03	1·68	2·45	2·57	2·17	2·98	26·6	0·28
Marine-origin *n*-3 PUFA^*^	% energy	0·19	0·11	0·29	0·26	0·19	0·36	36·8	0·28	g/d	0·48	0·27	0·74	0·64	0·45	0·89	33·3	0·25
EPA	% energy	0·06	0·03	0·09	0·08	0·06	0·12	49·4	0·28	g/d	0·14	0·07	0·24	0·20	0·14	0·30	43·3	0·25
*n*-3 DPA	% energy	0·02	0·02	0·03	0·03	0·02	0·04	23·6	0·24	g/d	0·06	0·04	0·08	0·07	0·05	0·09	20·0	0·22
DHA	% energy	0·11	0·06	0·17	0·15	0·11	0·21	36·4	0·28	g/d	0·28	0·15	0·43	0·37	0·27	0·50	32·1	0·25
*α*-linolenic acid	% energy	0·59	0·49	0·68	0·74	0·63	0·87	25·4	0·31	g/d	1·44	1·20	1·69	1·80	1·55	2·09	25·0	0·29
Cholesterol	mg/1000 kcal	160	137	188	174	138	205	8·8	0·36	mg/d	355	298	419	378	300	461	6·7	0·36
Carbohydrate	% energy	55·1	52·2	58·2	57·3	53·2	61·4	4·0	0·29	g/d	294	279	311	306	282	326	3·9	0·27
Added sugars	% energy	6·3	4·3	8·0	7·3	5·2	9·6	16·4	0·44	g/d	34·7	23·5	45·5	40·3	28·2	52·5	16·2	0·43
Total dietary fibre	g/1000 kcal	5·2	4·5	5·9	5·3	4·5	6·2	1·0	0·37	g/d	11·6	10·1	13·2	11·4	9·8	13·5	–1·0	0·30
Soluble dietary fibre	g/1000 kcal	1·3	1·1	1·5	1·3	1·0	1·6	1·6	0·33	g/d	2·8	2·4	3·2	2·8	2·3	3·4	0·7	0·25
Insoluble dietary fibre	g/1000 kcal	3·8	3·3	4·3	3·8	3·3	4·5	1·6	0·38	g/d	8·3	7·3	9·4	8·3	7·2	9·7	0·0	0·33
Retinol	μg/1000 kcal	110	90	132	154	118	214	39·8	0·31	μg/d	246	197	295	341	262	471	39·0	0·32
Vitamin A (retinol equivalent)^†^	μg/1000 kcal	225	187	274	281	218	392	24·9	0·35	μg/d	501	420	606	613	475	872	22·3	0·34
*α*-carotene	μg/1000 kcal	269	178	391	162	108	260	–39·7	0·24	μg/d	598	414	845	344	232	580	–42·5	0·21
*β*-carotene	μg/1000 kcal	1150	808	1521	1207	836	1724	4·9	0·34	μg/d	2625	1817	3322	2639	1832	3761	0·5	0·33
*β*-carotene equivalent^‡^	μg/1000 kcal	1373	956	1777	1374	974	1945	0·1	0·34	μg/d	3105	2136	3916	3009	2099	4310	–3·1	0·33
Cryptoxanthin	μg/1000 kcal	45	21	147	107	56	183	136·1	0·37	μg/d	99	46	335	247	117	399	148·6	0·36
Vitamin D	μg/1000 kcal	3·1	2·3	4·0	4·8	3·5	6·7	55·0	0·18	μg/d	6·8	4·9	8·9	10·7	7·3	14·6	55·7	0·16
*α*-tocopherol	mg/1000 kcal	4·1	3·6	4·5	3·7	3·2	4·2	–9·6	0·32	mg/d	8·9	7·8	10·1	8·1	7·1	9·3	–10·0	0·32
Vitamin K	μg/1000 kcal	82	66	103	110	80	149	33·8	0·40	μg/d	180	143	229	241	175	326	33·5	0·40
Thiamine	mg/1000 kcal	0·5	0·5	0·6	0·4	0·4	0·4	–21·6	0·16	mg/d	1·1	1·0	1·3	0·9	0·8	1·0	–22·3	0·17
Riboflavin	mg/1000 kcal	0·6	0·5	0·7	0·7	0·6	0·8	18·6	0·43	mg/d	1·3	1·2	1·5	1·5	1·3	1·8	14·4	0·44
Niacin	mg/1000 kcal	7·3	6·5	8·2	6·8	5·9	7·9	–6·6	0·16	mg/d	16·0	14·1	18·1	14·8	12·7	17·2	–7·6	0·17
Vitamin B_6_	mg/1000 kcal	0·6	0·5	0·7	0·6	0·5	0·6	–5·2	0·26	mg/d	1·3	1·1	1·4	1·2	1·1	1·4	–5·5	0·25
Vitamin B_12_	μg/1000 kcal	2·3	1·7	3·0	3·2	2·4	4·3	39·0	0·35	μg/d	5·0	3·8	6·7	6·9	5·2	9·2	37·8	0·34
Folate	μg/1000 kcal	123	106	141	149	122	178	21·4	0·38	μg/d	270	233	314	328	265	392	21·5	0·35
Pantothenic acid	mg/1000 kcal	2·9	2·6	3·1	3·3	3·0	3·7	16·4	0·49	mg/d	6·4	5·8	6·9	7·3	6·6	8·2	14·0	0·49
Vitamin C	mg/1000 kcal	39	30	48	49	37	62	24·9	0·38	mg/d	85	66	105	107	80	135	25·6	0·36
Na	mg/1000 kcal	1744	1566	1916	1704	1502	1963	–2·3	0·25	mg/d	3880	3474	4266	3738	3300	4253	–3·7	0·21
Potassium	mg/1000 kcal	1098	968	1217	1136	990	1305	3·4	0·43	mg/d	2461	2169	2714	2473	2156	2848	0·5	0·41
Ca	mg/1000 kcal	268	214	328	308	249	388	14·9	0·56	mg/d	599	478	713	671	545	854	11·9	0·57
Mg	mg/1000 kcal	108	98	120	116	102	130	6·8	0·45	mg/d	239	218	266	253	223	283	6·2	0·41
Phosphorus	mg/1000 kcal	513	470	565	542	487	618	5·7	0·53	mg/d	1145	1038	1247	1179	1065	1340	3·0	0·51
Fe	mg/1000 kcal	3·2	2·9	3·5	3·6	3·2	4·1	14·9	0·33	mg/d	7·0	6·3	7·8	7·9	6·9	9·0	13·0	0·30
Zn	mg/1000 kcal	4·4	4·1	4·7	4·4	4·0	4·7	–0·5	0·35	mg/d	9·7	8·9	10·3	9·5	8·8	10·2	–1·6	0·34
Cu	mg/1000 kcal	0·5	0·5	0·5	0·6	0·5	0·6	14·0	0·43	mg/d	1·1	1·0	1·2	1·2	1·1	1·4	12·7	0·44
Mn	mg/1000 kcal	1·3	1·2	1·5	1·7	1·4	2·0	27·6	0·27	mg/d	3·0	2·6	3·4	3·8	3·0	4·4	29·8	0·29

P25, 25th percentile; P75, 75th percentile.

* Sum of EPA, *n*-3 DPA and DHA.

† Sum of retinol, *β*-carotene/12, *α*-carotene/24 and cryptoxanthin/24.

‡ Sum of *β*-carotene, *α*-carotene/2 and cryptoxanthin/2.

§ Percentage differences: (BDHQ15y – 8-day DR)/8-day DR × 100 (%).

**Table 3.** Median estimates of daily energy and energy-adjusted nutrient intakes using the density and residual methods from the 8-day weighed dietary records (DR) and the brief-type diet history questionnaire for Japanese adolescents and children (BDHQ15y) in 412 Japanese girls aged 6–17 years: percentage differences in median intakes and Spearman’s rank correlations coefficients (CCs)

	Energy-adjusted by the density method									Energy-adjusted by the residual method								
	Unit	8-day DR			BDHQ15y			Median difference (%)^§^	Spearman’s rank CC	Unit	8-day DR			BDHQ15y			Median difference (%)^§^	Spearman’s rank CC
		Median	P25	P75	Median	P25	P75				Median	P25	P75	Median	P25	P75		
Energy	–	–		–	–		–		–	kcal/d	1898	1658	2120	1876	1590	2247	–1·1	0·41
Protein	% energy	14·3	13·3	15·2	14·1	12·8	15·5	–1·5	0·37	g/d	78·2	73·0	82·0	75·3	69·7	81·9	–3·7	0·35
Fat	% energy	31·8	29·3	33·9	30·6	27·6	33·3	–3·9	0·19	g/d	77·1	72·0	82·0	72·0	65·6	78·6	–6·6	0·18
SFA	% energy	10·3	9·3	11·3	9·5	8·2	11·0	–7·7	0·28	g/d	24·8	22·6	26·8	22·4	19·9	25·9	–9·7	0·26
MUFA	% energy	11·9	10·6	12·9	10·6	9·4	11·7	–10·6	0·19	g/d	29·3	26·7	31·5	24·6	22·3	27·5	–15·9	0·18
PUFA	% energy	5·9	5·3	6·5	6·4	5·7	7·3	8·9	0·19	g/d	14·2	12·9	15·5	15·0	13·3	17·0	5·8	0·16
*n*-6 PUFA	% energy	4·6	4·0	5·1	5·4	4·7	6·1	18·0	0·19	g/d	11·1	9·9	12·2	12·6	11·2	14·3	13·1	0·17
*n*-3 PUFA	% energy	0·89	0·72	1·06	1·12	0·95	1·29	25·8	0·18	g/d	2·08	1·75	2·47	2·59	2·26	3·02	24·5	0·17
Marine-origin *n*-3 PUFA^*^	% energy	0·21	0·12	0·32	0·28	0·20	0·38	33·3	0·27	g/d	0·48	0·30	0·71	0·67	0·51	0·88	39·6	0·26
EPA	% energy	0·06	0·03	0·10	0·09	0·06	0·12	37·1	0·28	g/d	0·14	0·08	0·22	0·21	0·16	0·29	49·7	0·27
*n*-3 DPA	% energy	0·02	0·02	0·03	0·03	0·02	0·04	25·9	0·28	g/d	0·06	0·04	0·08	0·07	0·05	0·09	24·7	0·28
DHA	% energy	0·12	0·07	0·19	0·16	0·12	0·22	33·3	0·26	g/d	0·27	0·17	0·42	0·39	0·30	0·50	44·4	0·26
*α*-linolenic acid	% energy	0·62	0·52	0·72	0·77	0·67	0·91	24·2	0·22	g/d	2·08	1·75	2·47	2·59	2·26	3·02	24·5	0·19
Cholesterol	mg/1000 kcal	171	140	201	188	147	225	9·9	0·33	mg/d	369	314	429	398	321	477	7·8	0·31
Carbohydrate	% energy	53·9	51·3	56·5	55·4	52·1	58·5	2·7	0·19	g/d	291	278	303	301	282	315	3·6	0·18
Added sugars	% energy	6·5	4·8	8·3	7·4	5·4	9·1	14·0	0·43	g/d	35·0	27·4	43·9	41·8	32·5	49·9	19·2	0·37
Total dietary fibre	g/1000 kcal	5·7	5·1	6·4	5·7	5·0	6·7	0·9	0·32	g/d	12·0	10·8	13·3	12·2	10·8	14·2	1·9	0·35
Soluble dietary fibre	g/1000 kcal	1·4	1·2	1·6	1·4	1·2	1·7	2·9	0·25	g/d	2·9	2·6	3·3	3·0	2·6	3·7	4·8	0·30
Insoluble dietary fibre	g/1000 kcal	4·1	3·6	4·5	4·2	3·7	4·8	2·0	0·35	g/d	8·7	7·7	9·6	8·9	7·8	10·3	2·4	0·37
Retinol	μg/1000 kcal	117	98	141	153	126	198	30·3	0·21	μg/d	251	215	295	352	297	431	40·0	0·18
Vitamin A (retinol equivalent)^†^	μg/1000 kcal	251	205	290	305	243	409	21·4	0·23	μg/d	520	439	607	654	548	864	25·8	0·24
*α*-carotene	μg/1000 kcal	295	211	402	195	122	298	–33·9	0·30	μg/d	594	434	798	365	267	624	–38·5	0·29
*β*-carotene	μg/1000 kcal	1301	971	1678	1402	953	2041	7·8	0·29	μg/d	2675	2083	3292	2968	2102	4087	11·0	0·29
*β*-carotene equivalent^‡^	μg/1000 kcal	1511	1181	1972	1579	1119	2261	4·5	0·30	μg/d	3139	2462	3882	3360	2474	4592	7·0	0·30
Cryptoxanthin	μg/1000 kcal	80	25	165	143	71	235	77·6	0·44	μg/d	176	73	346	318	199	506	80·6	0·45
Vitamin D	μg/1000 kcal	3·2	2·5	4·3	4·9	3·5	6·5	53·8	0·25	μg/d	6·8	5·4	8·8	11·5	8·7	14·2	69·5	0·26
*α*-tocopherol	mg/1000 kcal	4·4	3·9	4·9	4·0	3·6	4·5	–7·4	0·08	mg/d	9·4	8·4	10·5	8·5	7·6	9·7	–8·9	0·10
Vitamin K	μg/1000 kcal	91	75	116	120	89	163	31·8	0·39	μg/d	197	166	243	256	197	335	29·9	0·40
Thiamine	mg/1000 kcal	0·5	0·5	0·6	0·4	0·4	0·5	–21·2	0·24	mg/d	1·1	1·0	1·2	0·9	0·8	1·0	–20·5	0·22
Riboflavin	mg/1000 kcal	0·6	0·6	0·7	0·7	0·7	0·8	16·1	0·31	mg/d	1·3	1·2	1·5	1·5	1·4	1·7	16·7	0·32
Niacin	mg/1000 kcal	7·4	6·5	8·5	7·3	6·4	8·5	–1·9	0·31	mg/d	16·3	14·7	18·4	15·5	13·9	17·6	–5·3	0·32
Vitamin B_6_	mg/1000 kcal	0·6	0·5	0·7	0·6	0·5	0·7	–3·3	0·35	mg/d	1·3	1·2	1·4	1·3	1·1	1·4	–3·1	0·34
Vitamin B_12_	μg/1000 kcal	2·3	1·7	3·2	3·4	2·6	4·3	45·0	0·21	μg/d	4·9	3·9	6·5	7·3	5·9	9·2	47·1	0·20
Folate	μg/1000 kcal	136	119	155	162	138	197	19·1	0·26	μg/d	291	255	327	338	292	402	16·0	0·31
Pantothenic acid	mg/1000 kcal	3·0	2·7	3·2	3·4	3·1	3·8	15·6	0·39	mg/d	6·4	5·9	6·8	7·4	6·8	8·0	15·0	0·39
Vitamin C	mg/1000 kcal	43	35	55	56	44	71	28·3	0·35	mg/d	92	76	114	116	96	145	26·7	0·39
Sodium	mg/1000 kcal	1832	1671	2057	1836	1626	2039	0·2	0·24	mg/d	3906	3592	4319	3857	3466	4301	–1·3	0·23
Potassium	mg/1000 kcal	1171	1048	1274	1194	1041	1387	2·0	0·37	mg/d	2490	2270	2695	2550	2256	2883	2·4	0·37
Calcium	mg/1000 kcal	278	233	332	307	260	366	10·4	0·46	mg/d	581	505	670	668	587	776	14·8	0·45
Magnesium	mg/1000 kcal	114	104	126	120	107	133	4·8	0·38	mg/d	245	226	264	259	234	285	5·8	0·39
Phosphorus	mg/1000 kcal	529	488	571	558	500	614	5·6	0·38	mg/d	1138	1066	1212	1198	1093	1312	5·3	0·39
Iron	mg/1000 kcal	3·4	3·1	3·8	3·9	3·4	4·4	12·5	0·26	mg/d	7·3	6·7	8·0	8·2	7·4	9·3	11·7	0·26
Zinc	mg/1000 kcal	4·3	4·1	4·7	4·4	4·0	4·7	0·7	0·31	mg/d	9·6	9·0	10·2	9·4	8·8	10·1	–1·4	0·29
Copper	mg/1000 kcal	0·5	0·5	0·6	0·6	0·5	0·6	13·7	0·40	mg/d	1·1	1·0	1·2	1·3	1·2	1·4	12·5	0·40
Manganese	mg/1000 kcal	1·4	1·2	1·6	1·8	1·5	2·2	29·5	0·21	mg/d	3·1	2·8	3·5	3·8	3·2	4·4	23·1	0·25

SFA, saturated fatty acids; MUFA, monounsaturated fatty acids; PUFA, polyunsaturated fatty acids; EPA, eicosapentaenoic acid; DPA, docosapentaenoic acid; DHA, docosahexaenoic acid; P25, 25th percentile; P75, 75th percentile.

* Sum of EPA, *n*-3 DPA and DHA.

† Sum of retinol, *β*-carotene/12, *α*-carotene/24 and cryptoxanthin/24.

‡ Sum of *β*-carotene, *α*-carotene/2 and cryptoxanthin/2.

§ Percentage differences: (BDHQ15y – 8-day DR)/8-day DR × 100 (%).

Spearman’s rank correlation coefficients for energy intake between the BDHQ15y and 8-day DR were 0·67 for boys and 0·41 for girls. Among the forty-four nutrients examined, Spearman’s rank correlation coefficients in boys ranged from 0·16 (thiamine) to 0·56 (Ca) in the density model and from 0·16 (vitamin D) to 0·57 (Ca) in the residual model, with medians of 0·33 (25th–75th percentiles: 0·28–0·38) and 0·32 (25th–75th percentiles: 0·25–0·36), respectively. For girls, Spearman’s rank correlation coefficients ranged from 0·08 (*α*-tocopherol) to 0·46 (Ca) in the density model and from 0·10 (*α*-tocopherol) to 0·45 (Ca) in the residual model, with medians of 0·28 (25th–75th percentiles: 0·23–0·35) and 0·29 (25th–75th percentiles: 0·22–0·36), respectively.

Energy-adjusted food group intakes from the BDHQ15y and 8-day DR are shown in Table 4. Of the thirty-one food groups examined, only 11 (35 %) in boys and seven (23 %) in girls showed differences within 10 % between the two methods, while many food groups were under- or overestimated by more than 20 %. Food groups with the smallest median differences (%) included grains (2·2 % for boys and 3·9 % for girls), total vegetables (–6·7 % for boys and −5·2 % for girls), green and yellow vegetables (9·2 % for boys and 7·3 % for girls), dairy products (0·6 % for boys and −9·5 % for girls), full-fat milk (–8·8 % for boys and −4·2 % for girls) and SSB (–4·4 % for boys and −1·5 % for girls). Spearman’s rank correlation coefficients ranged from 0·13 for meat to 0·61 for dairy products in boys and from 0·10 for seasonings to 0·65 for dairy products in girls, with medians of 0·36 (25th–75th percentiles: 0·29–0·42) and 0·29 (25th–75th percentiles: 0·24–0·36), respectively.

**Table 4.** Median estimates of energy-adjusted food group intakes using the density method from the 8-day weighed dietary records (DR) and the brief-type diet history questionnaire for Japanese adolescents and children (BDHQ15y) and their correlations in Japanese children and adolescents aged 6–17 years: percentage differences in median intakes and Spearman’s rank correlations coefficients (CC)

	Boys (*n* 432)								Girls (*n* 412)							
	8-day DR			BDHQ15y			Median difference (%)^*^	Spearman’s rank CC	8-day DR			BDHQ15y			Median difference (%)^*^	Spearman’s rank CC
Food group (g/1000 kcal)	Median	P25	P75	Median	P25	P75			Median	P25	P75	Median	P25	P75		
Grains	225	203	256	230	191	275	2·2	0·35	212	188	237	220	181	251	3·9	0·25
Rice	157	130	189	183	141	236	16·4	0·36	138	116	166	169	124	207	22·5	0·29
Bread	20·6	12·1	28·9	13·8	7·9	26·1	–32·9	0·45	22·9	13·6	32·1	16·7	9·5	28·1	–27·1	0·36
Noodles	34·2	21·1	48·2	24·5	18·6	35·1	–28·4	0·22	34·5	18·6	52·0	27·2	18·5	38·2	–21·2	0·26
Potatoes	19·8	13·2	27·3	15·0	9·4	21·7	–24·2	0·27	22·6	16·0	30·9	16·5	9·9	24·1	–27·2	0·17
Pulses	15·7	9·9	22·8	21·3	13·4	31·6	35·8	0·30	17·5	10·8	25·9	22·1	13·7	33·5	26·1	0·31
Total vegetables	97·3	76·6	121·6	90·8	65·9	124·8	–6·7	0·40	110·0	86·6	137·8	104·3	80·7	143·3	–5·2	0·29
Green and yellow vegetables	28·1	18·5	38·5	30·7	20·4	44·7	9·2	0·43	33·4	23·2	45·0	35·8	24·4	52·2	7·3	0·28
Other vegetables	57·6	45·3	73·0	50·0	34·4	66·9	–13·1	0·32	65·4	48·9	81·4	56·4	41·1	79·1	–13·7	0·25
Pickled vegetables	0·7	0·1	1·8	0·8	0·0	3·3	10·3	0·26	0·7	0·1	2·1	1·1	0·0	3·5	51·4	0·22
Mushrooms	3·6	1·7	5·9	3·9	1·7	6·3	9·2	0·39	4·2	2·5	6·9	4·9	2·4	8·6	17·1	0·41
Seaweeds	2·7	1·5	5·1	3·4	1·7	5·9	23·1	0·31	3·6	1·7	6·8	4·0	1·7	6·7	9·3	0·19
Fruits	19·8	7·6	36·0	22·7	10·9	41·9	14·6	0·52	24·0	11·4	44·2	31·4	14·6	54·8	30·8	0·55
Fish and shellfish	17·6	11·0	26·0	25·1	17·9	35·6	42·6	0·33	20·3	12·8	28·7	28·5	20·0	38·8	40·1	0·26
Meat	54·3	44·2	65·5	35·3	28·8	45·6	–35·0	0·13	49·9	39·6	61·9	37·6	29·0	48·5	–24·5	0·27
Eggs	19·2	14·0	26·9	18·6	10·9	25·7	–3·1	0·42	21·5	15·3	29·3	18·8	11·5	28·1	–12·6	0·36
Dairy products	92·5	53·9	140·4	93·0	56·9	155·0	0·6	0·61	92·2	53·7	133·1	83·5	47·0	126·4	–9·5	0·65
Full-fat milk	71·7	39·6	113·2	65·4	31·2	118·0	–8·8	0·56	62·5	32·1	103·9	59·9	12·0	94·7	–4·2	0·64
Low-fat milk	0·1	0·0	0·4	0·0	0·0	0·0	–100	0·27	0·1	0·0	0·4	0·0	0·0	0·0	–100	0·17
Yoghurt	9·7	0·0	19·8	10·5	3·2	24·2	7·8	0·56	10·0	2·6	23·7	7·7	3·2	23·2	–22·9	0·48
Cheese	1·9	0·8	3·6	1·9	1·0	4·6	1·1	0·36	2·3	0·7	4·1	1·8	1·0	4·5	–20·7	0·32
Fat and oil	5·8	4·4	7·0	6·3	5·0	7·9	9·0	0·18	6·2	4·7	7·7	7·0	5·4	8·8	13·8	0·18
Sugar and confectionaries	21·7	12·8	34·6	37·4	24·6	52·8	72·5	0·39	28·0	18·0	39·0	41·8	28·5	59·0	49·4	0·35
Sugar	4·1	2·8	5·7	1·7	1·1	2·7	–57·6	0·15	4·6	3·2	6·5	2·0	1·3	3·2	–56·2	0·14
Confectionaries	17·6	8·7	29·6	35·3	22·1	50·0	100·9	0·38	23·0	13·1	32·6	39·2	24·5	56·5	70·4	0·35
Beverages	300	220	404	345	254	465	15·2	0·30	300	210	400	366	275	474	22·0	0·17
Water	37·6	8·9	96·2	72·9	9·5	196·2	93·6	0·30	39·5	10·5	100·1	60·4	5·7	199·0	53·0	0·27
Tea	148	74	242	179	63	264	20·8	0·42	173	87	263	216	89	296	24·8	0·36
Fruit and vegetable juice	2·3	0·0	15·9	9·4	0·0	31·3	300	0·39	0·2	0·0	15·6	8·8	0·0	27·4	4789	0·44
Sugar-sweetened beverages	44·7	17·1	88·4	42·7	17·8	81·6	–4·4	0·46	29·0	5·7	70·7	28·5	10·6	54·9	–1·5	0·43
Seasonings	47·4	33·6	64·9	9·2	7·1	12·0	–80·5	0·15	50·9	37·6	67·6	10·2	7·8	12·6	–79·9	0·10

P25, 25th percentile; P75, 75th percentile.

* Percentage differences: (BDHQ15y – 8-day DR)/8-day DR × 100 (%).

**Figure 2.**
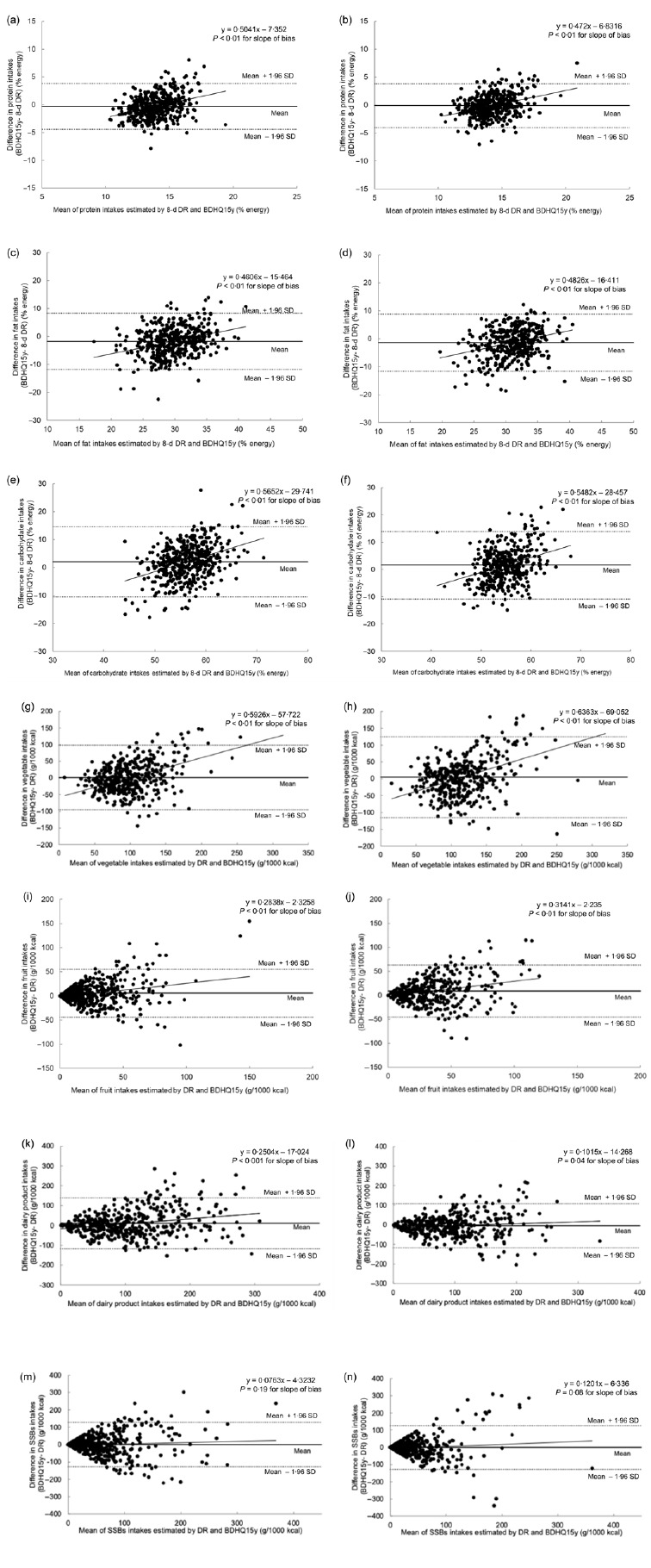
Bland–Altman plots of agreement between the brief-type diet history questionnaire for Japanese children and adolescents (BDHQ15y) and 8-day dietary records (DR) among 432 boys (a) protein, (c) fat, (e) carbohydrate, (g) total vegetables, (i) fruits, (k) dairy products and (m) sugar-sweetened beverages) and 412 girls (b) protein, (d) fat, (f) carbohydrate, (h) total vegetables, (j) fruits, (l) dairy products and (n) sugar-sweetened beverages) aged 6–17 years.

Bland–Altman plots were also used to assess agreement between the two methods for selected variables, namely, protein, total fat, carbohydrate, total vegetables, fruit, dairy products and SSB (Figure 2). The limits of agreement (mean (sd 1·96)) were relatively wide for all variables examined, indicating substantial discrepancies at the individual level. Regression lines for most variables examined were statistically significant, indicating a proportional bias in the BDHQ15y, with overestimation of intakes at higher consumption levels. In contrast, no significant linear trends were detected for SSB in boys or girls.

Additional analyses by sex and age groups (6–9, 10–14 and 15–17 years) revealed that, regardless of the energy-adjustment model, similar patterns of percentage differences in median intakes of nutrients and food groups (within 10 %) were observed across age groups for both sexes (online Supplementary Tables ST2–ST7). However, Spearman’s rank correlation coefficients for nutrients were generally lower in the 10- to 14-year age group than in the other age groups, with median values (from the density model results) of 0·28 in boys and 0·24 in girls, compared with 0·36 and 0·30 in the 6–9-year age group, respectively, and 0·37 and 0·34 in the 15–17-year age group, respectively. Notably, the 15- to 17-year age group had a higher number of nutrients and food groups with correlation coefficients exceeding 0·4 in both sexes than the other age groups.

## Discussion

To our knowledge, this is the first study to evaluate the relative validity of the BDHQ15y in assessing intakes of a broad range of nutrients and food groups. Using the 8-day DR as the reference method, our findings demonstrate that the BDHQ15y provides reasonable estimates of median intakes of several key nutrients and food groups, although its ability to accurately rank individuals differed considerably depending on the nutrient, food group and age group, with correlations generally lower in younger adolescents (10- to 14-year age group). These results suggest the potential of the BDHQ15y for large-scale dietary monitoring and public health research, particularly for assessing dietary intake trends of specific nutrients and food groups at the group level. However, its limitations, including variability in ranking ability, should be carefully considered, particularly when interpreting results for individual dietary assessment or specific sub-groups.

Unlike the adult versions of the DHQ and BDHQ in Japan^(25–29)^, which tend to underestimate dietary intake compared with DR, the BDHQ15y generally overestimated median intakes for most nutrients and food groups. This aligns with broader trends observed when adult-oriented dietary assessment tools were applied to children^(14–17,19–21)^. The BDHQ15y overestimated fifteen and fourteen nutrients in boys and girls, respectively, and underestimated two nutrients in both sexes, with all discrepancies exceeding 20 % relative to the 8-day DR. However, for key nutrients such as protein, fat, carbohydrate, dietary fibre and certain vitamins (e.g. niacin and vitamin B_6_) and minerals (e.g. Na, phosphorus and Zn), as well as food groups like grains, total vegetables, dairy products and SSB, the BDHQ15y provided reliable estimates within 10 % of the 8-day DR, suggesting its utility for population-level dietary monitoring. However, Bland–Altman analysis revealed greater variability at the individual level, with overestimations being more pronounced at higher intake levels. This may be due to the use of fixed portion sizes, which may lead frequent consumers to overestimate their intake if the portion sizes specified in the BDHQ15y are larger than those they actually consume. Therefore, caution should be exercised when interpreting individual-level data, especially for frequently consumed foods. Given that the portion sizes in the BDHQ15y were derived from the adult version of the BDHQ, which is based on recipe books for middle-aged women and adjusted using sex- and age-specific estimated energy requirement ratios^(28,29)^, future improvements to the BDHQ15y should focus on refining portion size estimates by updating them with current consumption data from the target population to more accurately reflect typical dietary intake.

The BDHQ15y demonstrated a limited ability to rank individuals by energy-adjusted nutrient intake, with median Spearman’s rank correlation coefficients (for results based on the density model) of 0·33 for boys and 0·28 for girls. These results are generally consistent with, although in some cases they are slightly lower than, those reported in a meta-analysis of dietary questionnaires in children and adolescents^(41–44)^. In particular, low correlations (< 0·3 or 0·4), which significantly attenuate the ability to detect associations in epidemiological studies^(6)^, were observed for several specific nutrients, including fat-related nutrients (e.g. *n*-3 PUFA, marine-origin *n*-3 PUFA, eicosapentaenoic acid, docosapentaenoic acid, docosahexaenoic acid and *α*-linolenic acid), certain vitamins (e.g. *α*-carotene, vitamin D and thiamine), as well as Na and Mn. These insufficient correlations likely reflect the inherent difficulty in accurately estimating nutrient intake from diverse food sources such as meat, fish, fats and oils and seasonings^(44)^. Additionally, the self-reporting nature of the BDHQ15y limits its ability to capture individual variations in fat preferences and cooking methods, which likely further contributes to these inaccuracies^(28,29,45)^. In contrast, stronger correlations were observed for certain nutrients, with correlations exceeding 0·5 for Ca and phosphorus in boys and exceeding 0·4 for several nutrients, including added sugar, vitamin K, Ca and Cu, in both sexes. Additionally, boys had higher correlations (> 0·4) for riboflavin, pantothenic acid, potassium, Mg and phosphorus, while girls showed a similar trend for cryptoxanthin. In light of these findings, the limited ability of the BDHQ15y to reliably rank nutrient intakes could hinder the identification of meaningful associations between dietary factors and health outcomes for many nutrients. While the BDHQ15y may be useful for broad population-level dietary monitoring, its application in epidemiological studies investigating specific nutrient-health relationships in children and adolescents should be approached with caution. Future research should prioritise biomarker-based validation studies employing appropriate biological samples (e.g. erythrocyte membrane fatty acids) across diverse populations to assess the accuracy of the BDHQ15y, particularly for nutrients that are prone to misestimation due to the self-reporting nature of this tool, such as various types of fatty acids, vitamin A and vitamin D. Such efforts would provide a more accurate evaluation of its reliability and enhance its applicability in nutritional epidemiology.

Despite lower correlations for many nutrients, it is noteworthy that the BDHQ15y achieved correlations exceeding 0·4 for ten and seven food groups in boys and girls, respectively. Common to both sexes, these included fruit, dairy products, full-fat milk, yoghurt and SSB. For boys, additional food groups surpassing this threshold included bread, total vegetables, green and yellow vegetables and tea. For girls, these food groups included mushrooms and fruit and vegetable juices. Notably, stronger correlations exceeding 0·5 were consistently observed for fruit, dairy products and full-fat milk in both sexes. These food groups play a crucial role in understanding dietary patterns in children and adolescents, with direct implications for dietary intake and associated health outcomes^(41)^. These findings highlight the utility of the BDHQ15y as a valuable tool for ranking individuals according to the intake of key food components in epidemiological studies, particularly those investigating the relationships between diet and health outcomes in younger populations.

Age-related differences in the validity of the BDHQ15y were observed, with the 10–14-year age group showing lower correlation coefficients than the other age groups. This may reflect the transitional nature of this developmental stage, during which younger adolescents gain greater independence in food choices and are influenced by social factors such as peer pressure, leading to more varied and less consistent dietary patterns^(9–12)^. Additionally, cognitive and memory-related challenges specific to this age group may contribute to the lower accuracy of dietary reporting^(11,42)^. In contrast, younger children (6–9 years) benefit from greater parental involvement, leading to more consistent reporting, while older adolescents (15–17 years) show improved accuracy due to cognitive maturity and more stable eating patterns. These findings highlight the need for careful interpretation of dietary assessment results among younger adolescents (i.e. 10–14-year age group) and emphasise the importance of developing age-appropriate tools. Strategies such as encouraging parental involvement and tailoring methods to this developmental stage could help address these challenges and improve the validity of dietary assessments.

Our study has several strengths, including a large, nationwide sample of Japanese school-aged children and adolescents (6–17 years), comprising both boys and girls. This broad age range and inclusion of both sexes enable comprehensive analysis of dietary intake across various developmental stages, enhancing the relevance and generalisability of our findings to the wider population of Japanese children and adolescents. Despite these strengths, several limitations should be considered. First, the participants were volunteers rather than a randomly selected sample, potentially leading to a group of participants with greater health consciousness due to the demanding nature of the 8-day DR. Second, use of the 8-day weighed DR as the reference method has inherent limitations, including errors from underreporting, over-reporting and social desirability bias, as well as potential changes in dietary behaviours during recording^(6)^. Unlike memory-based dietary assessments, the weighed DR does not rely on recall, which reduces some sources of error. However, shared biases between the DR and BDHQ15y, both of which involve self-reported data, may still affect their comparability^(6,42)^. Third, the BDHQ15y assessed dietary intake over the previous month, whereas the DR was conducted over the course of a year. This temporal discrepancy constitutes a methodological limitation, particularly in a population experiencing rapid developmental and behavioural changes. Additionally, seasonal variation in food consumption^(46)^ and age-related shifts in dietary habits^(9,11)^ contribute to differences between the two methods. Therefore, discrepancies in estimated intake may reflect both measurement error and genuine dietary changes over time, warranting cautious interpretation of the relative validity results for the BDHQ15y obtained in this study. Fourth, alcoholic beverages were not assessed in the BDHQ15y due to legal constraints and the difficulty of obtaining reliable self-reports from minors, which may be affected by social desirability bias. While alcohol use is generally low in this age group^(47)^, previous research suggests that it may be more prevalent among certain subgroups of older adolescents^(48)^. Consequently, the omission of alcoholic beverages may compromise the completeness of dietary assessment in this population. Finally, while half the participants recorded dietary intake only on weekdays, the remaining half recorded dietary intake on both weekdays and weekend days. Notably, four of the eight recorded days were weekend days, potentially overrepresenting weekend dietary habits. However, sensitivity analyses showed no significant differences between the two groups (data not shown), indicating that variations in recording days did not substantially affect the relative validity of BDHQ15y estimates.

In conclusion, the BDHQ15y demonstrates reasonable relative validity to assess the intakes of key nutrients and food groups in Japanese children and adolescents at the group level, but not at the individual level. While the BDHQ15y is effective for large-scale dietary monitoring, its ability to accurately rank individual intakes is limited, especially in younger adolescents. These findings highlight the need for caution when interpreting individual-level data. Future research should focus on refining portion sizes and conducting biomarker-based validation studies to more accurately assess the validity of the BDHQ15y. This would improve the evaluation of its validity and broaden its applicability in nutritional epidemiological studies. Furthermore, considering identified methodological limitations, developing and validating dietary questionnaires tailored to developmental stages is essential to better capture age-specific dietary behaviours and support epidemiological studies on diet and health.

## Supporting information

Okubo et al. supplementary material Okubo et al. supplementary material

## Supplementary material

For supplementary material/s referred to in this article, please visit https://doi.org/10.1017/S0007114525104042
