# Supplementary material for: Relative validity of food and nutrient intakes derived from a brief-type diet history questionnaire for Japanese children and adolescents
Source: Br J Nutr. 2025 Aug 26;134(5):425–39. doi: 10.1017/S0007114525104042 (PMC12580970; doi:10.1017/S0007114525104042)
Supplement: Okubo et al. supplementary material [file S0007114525104042sup001.docx]

**Supplemental Material**

**Title:** Relative validity of food and nutrient intakes derived from a brief-type diet history questionnaire for Japanese children and adolescents (BDHQ15y)

**Authors:** Hitomi Okubo^1^, Ryoko Tajima^1^, Nana Shinozaki^2^, Shizuko Masayasu^3^, Satoshi Sasaki^2^, Kentaro Murakami^2^

**Affiliations:**

^1^Department of Nutritional Epidemiology and Behavioural Nutrition, Graduate School of Medicine, The University of Tokyo, 7-3-1 Hongo, Bunkyo-ku, Tokyo 113-0033, Japan.

^2^Department of Social and Preventive Epidemiology, School of Public Health, The University of Tokyo, 7-3-1 Hongo, Bunkyo-ku, Tokyo 113-0033, Japan.

^3^Ikurien-Naka, 3799-6 Sugaya, Naka-shi, Ibaraki 311-0105, Japan.

**Supplemental Table 1**. Definitions of food groups in the brief-type diet history questionnaire for Japanese adolescents and children (BDHQ15y).

| Food groups | Food item in the BDHQ15y | *n* |
| --- | --- | --- |
| Grains |  |  |
| Rice | Well-milled rice; well-milled rice mixed with barley, well-milled rice with germ and brown rice | 2 |
| Bread | Bread (including white bread, filled and stuffed bread and pastries) | 1 |
| Noodles | Buckwheat noodles; Japanese wheat noodles; instant noodles and Chinese noodles; spaghetti and macaroni | 4 |
| Potatoes | Potatoes (all varieties); French fries and crisps | 2 |
| Pulses | Tofu (i.e., soybean curd) and tofu products; natto | 2 |
| Total vegetables |  |  |
| Green and yellow vegetables | Green leafy vegetables including broccoli; carrots and pumpkins; tomatoes, boiled tomatoes and stewed tomatoes | 3 |
| Other vegetables | Raw vegetables used in salad (cabbage and lettuce); cabbage and Chinese cabbage; radishes and turnips; other root vegetables (onions, burdock and lotus root) | 4 |
| Pickled vegetables | Salted green and yellow vegetable pickles; other salted vegetable pickles (excluding salted pickled plum) | 2 |
| Mushrooms | Mushrooms (all varieties) | 1 |
| Seaweeds | Seaweed (all varieties) | 1 |
| Fruit | Citrus fruits including oranges; strawberries, persimmons and kiwi fruit; other fruits | 3 |
| Fish and shellfish | Dried fish and salted fish (including salted mackerel, salted salmon and dried horse mackerel); small fish with bones; canned tuna; oily fish (including sardines, mackerel, saury, amberjack, herring, eel and fatty tuna); non-oily fish (including salon, trout, white meat fish, freshwater fish and bonito); squid, octopus, shrimp and clam; fish paste products | 7 |
| Meat | Chicken (including ground chicken); pork and beef (including ground pork and beef); liver; ham, sausages and bacon | 4 |
| Eggs | Eggs | 1 |
| Dairy products |  |  |
| Full-fat milk | Full-fat milk | 1 |
| Low-fat milk | Low-fat milk | 1 |
| Yoghurt | Yoghurt (including drink types) | 1 |
| Cheese | Cheese | 1 |
| Fats and oils | Butter; margarine; oil used during cooking | 3 |
| Sugar and confectionaries |  |  |
| Sugar | Jam; sugar used during cooking | 2 |
| Confectionaries | Cakes, cookies and biscuits; Japanese sweets; rice crackers, rice cakes and Japanese-style pancakes; savoury snacks; chocolates; ice cream; nutritional snacks | 7 |
| Beverages |  |  |
| Water | Water | 1 |
| Tea | Green tea; black tea and oolong tea | 2 |
| Fruit and vegetable juice | Pure fruit juice and vegetable juice (100%) | 1 |
| Sugar-sweetened beverages | Cola and sweetened soft drinks (including sports drinks and fruit drinks excluding 100%); coffee; lactic acid bacteria beverages; energy drinks | 4 |
| Seasoning | Noodle soup; soy sauce; mayonnaise; ketchup; table sauce and soy sauce; table salt; miso for miso soup | 7 |

**Table ST2.** Median estimates of daily energy and energy-adjusted nutrient intakes using the density method from the 8-day weighed dietary record (DR) and the brief-type diet history questionnaire for Japanese adolescents and children (BDHQ15y) in 432 Japanese boys aged 6–17 years: percentage differences in median intakes and Spearman’s rank correlations coefficients (CCs), according to the age group.

|  |  | 6–9 years (*n* 126) | | | | | | | |  | 10–14 years (*n* 184) | | | | | | | |  | 15–17 years (*n* 122) | | | | | | | |
| --- | --- | --- | --- | --- | --- | --- | --- | --- | --- | --- | --- | --- | --- | --- | --- | --- | --- | --- | --- | --- | --- | --- | --- | --- | --- | --- | --- |
|  | Unit | 8-day DR | | | BDHQ15y | | | Median difference (%) ^§^ | CC |  | 8-day DR | | | BDHQ15y | | | Median difference (%) ^§^ | CC |  | 8-day DR | | | BDHQ15y | | | Median difference (%) ^§^ | CC |
|  |  | Median | P25 | P75 | Median | P25 | P75 |  |  |  | Median | P25 | P75 | Median | P25 | P75 |  |  |  | Median | P25 | P75 | Median | P25 | P75 |  |  |
| Protein | % energy | 14.2 | 13.2 | 15.0 | 14.2 | 12.8 | 15.6 | -0.1 | 0.40 |  | 14.1 | 13.3 | 15.1 | 13.4 | 12.5 | 15.2 | -4.8 | 0.19 |  | 13.9 | 12.7 | 14.8 | 13.3 | 12.0 | 14.6 | -4.4 | 0.43 |
| Fat | % energy | 31.2 | 28.7 | 33.4 | 29.7 | 26.4 | 32.6 | -4.8 | 0.33 |  | 30.7 | 28.2 | 33.2 | 29.0 | 25.3 | 32.3 | -5.4 | 0.22 |  | 30.2 | 27.7 | 33.0 | 28.3 | 24.6 | 31.6 | -6.5 | 0.43 |
| SFA | % energy | 10.4 | 9.3 | 11.4 | 9.5 | 8.2 | 10.8 | -8.9 | 0.39 |  | 9.8 | 9.0 | 10.9 | 9.3 | 7.9 | 10.8 | -5.9 | 0.22 |  | 9.2 | 8.1 | 10.3 | 8.2 | 7.1 | 10.2 | -10.5 | 0.50 |
| MUFA | % energy | 11.5 | 10.4 | 12.4 | 10.2 | 8.9 | 11.1 | -11.9 | 0.26 |  | 11.5 | 10.5 | 12.6 | 9.8 | 8.5 | 11.0 | -14.4 | 0.19 |  | 11.8 | 10.7 | 13.2 | 9.9 | 8.2 | 10.9 | -16.1 | 0.38 |
| PUFA | % energy | 5.6 | 5.0 | 6.1 | 6.2 | 5.5 | 7.1 | 9.6 | 0.30 |  | 5.6 | 5.1 | 6.3 | 6.0 | 5.3 | 6.7 | 6.6 | 0.28 |  | 5.5 | 4.9 | 6.2 | 6.1 | 5.2 | 6.7 | 10.8 | 0.28 |
| n-6 PUFA | % energy | 4.3 | 3.8 | 4.9 | 5.2 | 4.5 | 6.0 | 20.7 | 0.25 |  | 4.3 | 4.0 | 4.9 | 5.1 | 4.4 | 5.6 | 17.1 | 0.23 |  | 4.4 | 3.9 | 4.8 | 5.2 | 4.4 | 5.7 | 18.6 | 0.19 |
| n-3 PUFA | % energy | 0.82 | 0.68 | 1.01 | 1.13 | 0.96 | 1.29 | 37.8 | 0.33 |  | 0.83 | 0.70 | 0.97 | 1.03 | 0.88 | 1.20 | 24.1 | 0.29 |  | 0.81 | 0.67 | 0.96 | 1.02 | 0.85 | 1.19 | 25.9 | 0.31 |
| Marine-origin n-3 PUFA* | % energy | 0.22 | 0.12 | 0.32 | 0.28 | 0.22 | 0.41 | 27.3 | 0.34 |  | 0.18 | 0.11 | 0.28 | 0.26 | 0.19 | 0.37 | 44.4 | 0.25 |  | 0.18 | 0.10 | 0.27 | 0.24 | 0.17 | 0.34 | 33.3 | 0.22 |
| EPA | % energy | 0.06 | 0.04 | 0.11 | 0.09 | 0.07 | 0.14 | 50.0 | 0.33 |  | 0.05 | 0.03 | 0.09 | 0.08 | 0.06 | 0.12 | 66.4 | 0.27 |  | 0.05 | 0.03 | 0.08 | 0.07 | 0.05 | 0.11 | 34.0 | 0.20 |
| n-3 DPA | % energy | 0.02 | 0.02 | 0.03 | 0.03 | 0.02 | 0.04 | 22.6 | 0.33 |  | 0.02 | 0.02 | 0.03 | 0.03 | 0.02 | 0.04 | 28.4 | 0.19 |  | 0.02 | 0.02 | 0.03 | 0.03 | 0.02 | 0.03 | 14.7 | 0.23 |
| DHA | % energy | 0.13 | 0.06 | 0.18 | 0.16 | 0.13 | 0.23 | 23.1 | 0.34 |  | 0.10 | 0.06 | 0.16 | 0.15 | 0.11 | 0.21 | 50.0 | 0.25 |  | 0.11 | 0.07 | 0.16 | 0.14 | 0.10 | 0.19 | 27.3 | 0.23 |
| α-linolenic acid | % energy | 0.59 | 0.47 | 0.69 | 0.77 | 0.66 | 0.89 | 30.5 | 0.27 |  | 0.59 | 0.52 | 0.69 | 0.73 | 0.62 | 0.84 | 23.7 | 0.36 |  | 0.58 | 0.49 | 0.67 | 0.73 | 0.62 | 0.84 | 25.9 | 0.30 |
| Cholesterol | mg/1000 kcal | 159 | 128 | 190 | 168 | 130 | 203 | 5.6 | 0.36 |  | 156 | 138 | 183 | 174 | 141 | 201 | 11.4 | 0.36 |  | 171 | 143 | 193 | 179 | 147 | 220 | 4.9 | 0.39 |
| Carbohydrate | % energy | 54.6 | 52.3 | 57.3 | 56.1 | 52.9 | 59.6 | 2.7 | 0.26 |  | 55.1 | 52.1 | 57.8 | 57.3 | 53.0 | 61.7 | 4.0 | 0.20 |  | 55.5 | 52.4 | 58.9 | 58.8 | 54.1 | 62.6 | 5.9 | 0.43 |
| Added sugar | % energy | 6.6 | 4.5 | 8.3 | 7.1 | 5.4 | 9.0 | 7.7 | 0.52 |  | 6.1 | 4.4 | 8.0 | 7.3 | 5.0 | 9.5 | 19.1 | 0.36 |  | 5.9 | 3.6 | 7.9 | 8.0 | 5.5 | 12.0 | 35.2 | 0.53 |
| Total dietary fibre | g/1000 kcal | 5.6 | 4.8 | 6.2 | 5.5 | 4.6 | 6.7 | -2.5 | 0.45 |  | 5.3 | 4.6 | 5.8 | 5.3 | 4.5 | 6.0 | -0.6 | 0.29 |  | 4.7 | 4.1 | 5.3 | 5.2 | 4.3 | 5.9 | 10.7 | 0.32 |
| Soluble dietary fibre | g/1000 kcal | 1.3 | 1.2 | 1.5 | 1.4 | 1.1 | 1.7 | 3.0 | 0.40 |  | 1.3 | 1.1 | 1.5 | 1.3 | 1.0 | 1.5 | -1.6 | 0.26 |  | 1.1 | 1.0 | 1.3 | 1.2 | 1.0 | 1.5 | 15.0 | 0.27 |
| Insoluble dietary fibre | g/1000 kcal | 4.0 | 3.5 | 4.5 | 4.0 | 3.4 | 4.8 | -1.5 | 0.51 |  | 3.8 | 3.4 | 4.2 | 3.8 | 3.3 | 4.3 | -0.5 | 0.28 |  | 3.5 | 2.9 | 3.9 | 3.7 | 3.2 | 4.3 | 7.2 | 0.32 |
| Retinol | μg/1000 kcal | 121 | 97 | 141 | 156 | 121 | 250 | 28.7 | 0.32 |  | 111 | 93 | 129 | 160 | 120 | 230 | 44.8 | 0.30 |  | 97 | 86 | 122 | 147 | 116 | 198 | 50.6 | 0.26 |
| Vitamin A (retinol equivalent)^†^ | μg/1000 kcal | 247 | 212 | 291 | 297 | 222 | 431 | 20.2 | 0.38 |  | 228 | 198 | 265 | 282 | 219 | 405 | 23.9 | 0.27 |  | 188 | 161 | 250 | 267 | 212 | 350 | 41.9 | 0.40 |
| α-carotene | μg/1000 kcal | 313 | 223 | 430 | 183 | 130 | 274 | -41.4 | 0.28 |  | 285 | 209 | 382 | 159 | 108 | 255 | -44.2 | 0.19 |  | 189 | 122 | 310 | 143 | 85 | 236 | -24.3 | 0.19 |
| β-carotene | μg/1000 kcal | 1350 | 949 | 1620 | 1246 | 883 | 1775 | -7.7 | 0.37 |  | 1166 | 842 | 1505 | 1168 | 833 | 1722 | 0.2 | 0.30 |  | 934 | 641 | 1366 | 1193 | 752 | 1650 | 27.7 | 0.37 |
| β-carotene equivalent^‡^ | μg/1000 kcal | 1595 | 1142 | 1950 | 1401 | 1034 | 2000 | -12.1 | 0.38 |  | 1403 | 1015 | 1748 | 1335 | 971 | 1954 | -4.9 | 0.30 |  | 1100 | 783 | 1610 | 1375 | 892 | 1857 | 25.0 | 0.37 |
| Cryptoxanthin | μg/1000 kcal | 66 | 26 | 172 | 120 | 66 | 179 | 82.3 | 0.41 |  | 40 | 20 | 151 | 94 | 50 | 173 | 132.8 | 0.35 |  | 34 | 19 | 120 | 107 | 60 | 199 | 211.0 | 0.32 |
| Vitamin D | μg/1000 kcal | 3.5 | 2.5 | 4.1 | 5.0 | 3.8 | 7.1 | 43.9 | 0.24 |  | 2.9 | 2.1 | 3.9 | 5.0 | 3.5 | 6.6 | 72.3 | 0.18 |  | 2.9 | 2.3 | 3.7 | 4.4 | 3.1 | 6.5 | 50.7 | 0.09 |
| α-tocopherol | mg/1000 kcal | 4.1 | 3.5 | 4.4 | 3.8 | 3.3 | 4.3 | -6.4 | 0.29 |  | 3.9 | 3.6 | 4.4 | 3.6 | 3.2 | 4.1 | -8.4 | 0.35 |  | 4.2 | 3.5 | 4.7 | 3.7 | 3.2 | 4.2 | -11.5 | 0.30 |
| Vitamin K | μg/1000 kcal | 83 | 68 | 105 | 114 | 87 | 151 | 36.9 | 0.34 |  | 82 | 65 | 102 | 108 | 76 | 148 | 32.6 | 0.37 |  | 79 | 67 | 103 | 109 | 84 | 148 | 37.7 | 0.52 |
| Thiamine | mg/1000 kcal | 0.5 | 0.5 | 0.6 | 0.4 | 0.4 | 0.5 | -21.2 | 0.16 |  | 0.5 | 0.5 | 0.6 | 0.4 | 0.4 | 0.4 | -20.0 | 0.06 |  | 0.5 | 0.5 | 0.6 | 0.4 | 0.3 | 0.4 | -22.0 | 0.27 |
| Riboflavin | mg/1000 kcal | 0.6 | 0.6 | 0.7 | 0.7 | 0.6 | 0.8 | 15.9 | 0.41 |  | 0.6 | 0.5 | 0.7 | 0.7 | 0.6 | 0.8 | 18.6 | 0.36 |  | 0.5 | 0.5 | 0.6 | 0.7 | 0.6 | 0.8 | 22.2 | 0.48 |
| Niacin | mg/1000 kcal | 7.3 | 6.4 | 8.0 | 7.2 | 6.1 | 8.5 | -0.6 | 0.26 |  | 7.2 | 6.6 | 8.2 | 6.6 | 5.8 | 7.5 | -8.3 | 0.00 |  | 7.3 | 6.4 | 8.4 | 6.5 | 5.8 | 7.8 | -9.9 | 0.29 |
| Vitamin B_6_ | mg/1000 kcal | 0.6 | 0.5 | 0.7 | 0.6 | 0.5 | 0.7 | -1.7 | 0.38 |  | 0.6 | 0.5 | 0.7 | 0.5 | 0.5 | 0.6 | -6.9 | 0.07 |  | 0.6 | 0.5 | 0.6 | 0.5 | 0.5 | 0.6 | -3.6 | 0.38 |
| Vitamin B_12_ | μg/1000 kcal | 2.6 | 1.8 | 3.4 | 3.5 | 2.6 | 4.6 | 38.3 | 0.34 |  | 2.3 | 1.8 | 3.0 | 3.2 | 2.4 | 4.3 | 36.1 | 0.33 |  | 2.0 | 1.5 | 2.7 | 2.9 | 2.3 | 3.7 | 40.9 | 0.28 |
| Folate | μg/1000 kcal | 131 | 113 | 141 | 155 | 127 | 188 | 18.8 | 0.41 |  | 120 | 106 | 140 | 146 | 117 | 174 | 22.0 | 0.31 |  | 115 | 100 | 141 | 147 | 124 | 175 | 28.3 | 0.44 |
| Pantothenic acid | mg/1000 kcal | 3.0 | 2.8 | 3.3 | 3.4 | 3.1 | 3.8 | 12.6 | 0.48 |  | 2.9 | 2.7 | 3.1 | 3.4 | 3.0 | 3.7 | 17.1 | 0.44 |  | 2.7 | 2.5 | 3.0 | 3.2 | 2.9 | 3.7 | 19.6 | 0.55 |
| Vitamin C | mg/1000 kcal | 40 | 32 | 49 | 51 | 41 | 61 | 26.2 | 0.42 |  | 39 | 31 | 48 | 46.9 | 34.6 | 61.1 | 19.4 | 0.29 |  | 36 | 29 | 46 | 49 | 36 | 63 | 35.0 | 0.45 |
| Sodium | mg/1000 kcal | 1811 | 1642 | 1942 | 1800 | 1557 | 2073 | -0.6 | 0.27 |  | 1744 | 1557 | 1924 | 1688 | 1463 | 1944 | -3.2 | 0.21 |  | 1660 | 1470 | 1846 | 1655 | 1468 | 1905 | -0.3 | 0.23 |
| Potassium | mg/1000 kcal | 1170 | 1064 | 1287 | 1194 | 1044 | 1337 | 2.1 | 0.47 |  | 1104 | 1012 | 1216 | 1120 | 982 | 1304 | 1.4 | 0.32 |  | 993 | 885 | 1137 | 1112 | 944 | 1289 | 12.0 | 0.50 |
| Calcium | mg/1000 kcal | 304 | 268 | 355 | 327 | 275 | 381 | 7.4 | 0.53 |  | 277 | 233 | 329 | 328 | 266 | 393 | 18.4 | 0.50 |  | 202 | 174 | 251 | 267 | 225 | 334 | 31.8 | 0.58 |
| Magnesium | mg/1000 kcal | 114 | 104 | 128 | 120 | 110 | 134 | 5.3 | 0.49 |  | 110 | 101 | 122 | 116 | 103 | 129 | 5.5 | 0.31 |  | 101 | 92 | 111 | 112 | 99 | 127 | 10.9 | 0.52 |
| Phosphorus | mg/1000 kcal | 541 | 506 | 589 | 556 | 505 | 627 | 2.7 | 0.52 |  | 514 | 476 | 566 | 543 | 492 | 629 | 5.5 | 0.44 |  | 480 | 440 | 523 | 518 | 457 | 583 | 7.7 | 0.59 |
| Iron | mg/1000 kcal | 3.2 | 2.9 | 3.6 | 3.7 | 3.3 | 4.3 | 13.9 | 0.37 |  | 3.2 | 2.9 | 3.5 | 3.5 | 3.1 | 4.0 | 11.1 | 0.22 |  | 3.1 | 2.8 | 3.4 | 3.7 | 3.2 | 4.1 | 18.9 | 0.41 |
| Zinc | mg/1000 kcal | 4.4 | 4.1 | 4.6 | 4.4 | 4.1 | 4.7 | 1.4 | 0.37 |  | 4.4 | 4.1 | 4.7 | 4.4 | 4.0 | 4.7 | -0.9 | 0.27 |  | 4.3 | 4.0 | 4.7 | 4.3 | 4.0 | 4.6 | -0.5 | 0.43 |
| Copper | mg/1000 kcal | 0.5 | 0.5 | 0.6 | 0.6 | 0.5 | 0.6 | 16.0 | 0.48 |  | 0.5 | 0.5 | 0.5 | 0.6 | 0.5 | 0.6 | 12.0 | 0.36 |  | 0.5 | 0.5 | 0.5 | 0.6 | 0.5 | 0.6 | 14.0 | 0.52 |
| Manganese | mg/1000 kcal | 1.3 | 1.2 | 1.5 | 1.8 | 1.4 | 2.1 | 34.4 | 0.29 |  | 1.3 | 1.2 | 1.5 | 1.7 | 1.4 | 2.0 | 28.0 | 0.17 |  | 1.4 | 1.3 | 1.7 | 1.7 | 1.4 | 2.0 | 21.1 | 0.41 |
|  |  |  |  |  |  |  |  |  |  |  |  |  |  |  |  |  |  |  |  |  |  |  |  |  |  |  |  |
| Median CC |  |  |  |  |  |  |  |  | 0.36 |  |  |  |  |  |  |  |  | 0.28 |  |  |  |  |  |  |  |  | 0.37 |

SFA, saturated fatty acids; MUFA, monounsaturated fatty acids; PUFA, polyunsaturated fatty acids; EPA, eicosapentaenoic acid; DPA, docosapentaenoic acid; DHA, docosahexaenoic acid; P25, 25th percentile; P75, 75th percentile.

^*^ Sum of EPA, n-3 DPA and DHA.

^†^ Sum of retinol, β-carotene/12, α-carotene/24 and cryptoxanthin/24.

^‡^ Sum of β-carotene, α-carotene/2 and cryptoxanthin/2.

^§^ Percentage differences: (BDHQ15y – 8-day DR)/8-day DR × 100 (%).

**Table ST3.** Median estimates of daily energy and energy-adjusted nutrient intakes using the residual method from the 8-day weighed dietary record (DR) and the brief-type diet history questionnaire for Japanese adolescents and children (BDHQ15y) in 432 Japanese boys aged 6–17 years: percentage differences in median intakes and Spearman’s rank correlations coefficients (CCs), according to the age group.

|  |  | 6–9 years (*n* 126) | | | | | | | |  | 10–14 years (*n* 184) | | | | | | | |  | 15–17 years (*n* 122) | | | | | | | |
| --- | --- | --- | --- | --- | --- | --- | --- | --- | --- | --- | --- | --- | --- | --- | --- | --- | --- | --- | --- | --- | --- | --- | --- | --- | --- | --- | --- |
|  | Unit | 8-day DR | | | BDHQ15y | | | Median difference (%) ^§^ | CC |  | 8-day DR | | | BDHQ15y | | | Median difference (%) ^§^ | CC |  | 8-day DR | | | BDHQ15y | | | Median difference (%) ^§^ | CC |
|  |  | Median | P25 | P75 | Median | P25 | P75 |  |  |  | Median | P25 | P75 | Median | P25 | P75 |  |  |  | Median | P25 | P75 | Median | P25 | P75 |  |  |
| Energy | kcal/d | 1814 | 1693 | 2047 | 1772 | 1491 | 2043 | -2.3 | 0.20 |  | 2446 | 2071 | 2879 | 2381 | 1937 | 2897 | -2.6 | 0.60 |  | 2986 | 2415 | 3394 | 2794 | 2344 | 3393 | -6.4 | 0.52 |
| Protein | g/d | 77.6 | 72.9 | 80.9 | 75.1 | 69.4 | 81.1 | -3.3 | 0.33 |  | 77.9 | 73.0 | 83.7 | 73.4 | 68.0 | 82.6 | -5.7 | 0.19 |  | 77.4 | 68.8 | 85.7 | 73.0 | 64.8 | 85.2 | -5.7 | 0.42 |
| Fat | g/d | 75.7 | 70.4 | 80.2 | 69.5 | 63.5 | 76.7 | -8.2 | 0.28 |  | 75.1 | 69.5 | 81.2 | 70.7 | 61.4 | 79.4 | -5.8 | 0.21 |  | 74.2 | 66.3 | 81.9 | 69.4 | 59.1 | 81.1 | -6.5 | 0.43 |
| SFA | g/d | 25.0 | 22.6 | 27.2 | 22.3 | 19.9 | 24.8 | -10.8 | 0.41 |  | 24.2 | 22.0 | 27.5 | 22.5 | 19.1 | 26.5 | -6.9 | 0.20 |  | 22.4 | 18.9 | 26.5 | 19.9 | 17.0 | 26.5 | -11.0 | 0.48 |
| MUFA | g/d | 28.4 | 26.1 | 30.2 | 23.7 | 21.3 | 25.9 | -16.6 | 0.23 |  | 28.0 | 25.2 | 30.8 | 23.7 | 20.8 | 26.8 | -15.3 | 0.19 |  | 28.5 | 25.0 | 32.3 | 24.8 | 19.9 | 28.5 | -13.2 | 0.36 |
| PUFA | g/d | 13.6 | 12.4 | 14.8 | 14.4 | 12.8 | 15.9 | 5.6 | 0.24 |  | 13.9 | 12.3 | 15.3 | 14.5 | 12.8 | 16.5 | 4.9 | 0.25 |  | 13.6 | 11.8 | 15.8 | 15.6 | 13.0 | 17.4 | 14.4 | 0.28 |
| n-6 PUFA | g/d | 10.5 | 9.7 | 11.6 | 12.2 | 10.8 | 13.4 | 15.8 | 0.19 |  | 10.6 | 9.6 | 11.8 | 12.4 | 10.8 | 13.9 | 16.8 | 0.21 |  | 10.7 | 9.0 | 12.3 | 13.1 | 10.7 | 14.5 | 23.0 | 0.19 |
| n-3 PUFA | g/d | 1.97 | 1.68 | 2.37 | 2.59 | 2.27 | 2.95 | 31.5 | 0.28 |  | 2.06 | 1.71 | 2.45 | 2.56 | 2.13 | 3.02 | 24.3 | 0.25 |  | 2.04 | 1.58 | 2.55 | 2.55 | 2.08 | 3.03 | 25.0 | 0.33 |
| Marine-origin n-3 PUFA* | g/d | 0.50 | 0.30 | 0.74 | 0.66 | 0.55 | 0.92 | 32.0 | 0.32 |  | 0.44 | 0.26 | 0.74 | 0.64 | 0.44 | 0.88 | 45.5 | 0.23 |  | 0.51 | 0.23 | 0.75 | 0.59 | 0.37 | 0.88 | 15.7 | 0.24 |
| EPA | g/d | 0.15 | 0.09 | 0.23 | 0.22 | 0.17 | 0.31 | 43.9 | 0.30 |  | 0.13 | 0.07 | 0.23 | 0.21 | 0.13 | 0.28 | 55.6 | 0.24 |  | 0.14 | 0.05 | 0.24 | 0.17 | 0.10 | 0.30 | 19.3 | 0.22 |
| n-3 DPA | g/d | 0.06 | 0.04 | 0.08 | 0.07 | 0.06 | 0.09 | 21.8 | 0.31 |  | 0.05 | 0.04 | 0.08 | 0.07 | 0.05 | 0.09 | 24.9 | 0.18 |  | 0.06 | 0.04 | 0.09 | 0.06 | 0.04 | 0.09 | 5.6 | 0.22 |
| DHA | g/d | 0.29 | 0.16 | 0.41 | 0.38 | 0.31 | 0.51 | 31.0 | 0.32 |  | 0.26 | 0.14 | 0.43 | 0.37 | 0.26 | 0.50 | 42.3 | 0.23 |  | 0.31 | 0.15 | 0.44 | 0.35 | 0.23 | 0.49 | 12.9 | 0.24 |
| α-linolenic acid | g/d | 1.43 | 1.19 | 1.64 | 1.77 | 1.55 | 2.01 | 23.8 | 0.21 |  | 1.45 | 1.25 | 1.71 | 1.77 | 1.51 | 2.09 | 22.1 | 0.33 |  | 1.43 | 1.09 | 1.73 | 1.86 | 1.52 | 2.16 | 30.1 | 0.28 |
| Cholesterol | mg/d | 349 | 295 | 405 | 353 | 292 | 434 | 1.2 | 0.35 |  | 346 | 298 | 406 | 379 | 308 | 458 | 9.6 | 0.33 |  | 386 | 306 | 451 | 404 | 319 | 501 | 4.8 | 0.40 |
| Carbohydrate | g/d | 292 | 283 | 307 | 306 | 291 | 320 | 4.5 | 0.19 |  | 295 | 277 | 310 | 304 | 279 | 328 | 3.2 | 0.19 |  | 295 | 275 | 317 | 307 | 277 | 333 | 4.1 | 0.41 |
| Added sugar | g/d | 35.2 | 26.3 | 43.3 | 41.6 | 33.3 | 49.6 | 18.4 | 0.48 |  | 34.1 | 24.0 | 45.5 | 38.2 | 25.1 | 52.1 | 11.9 | 0.31 |  | 32.8 | 17.4 | 47.8 | 40.6 | 21.9 | 69.0 | 23.8 | 0.55 |
| Total dietary fibre | g/d | 11.7 | 10.5 | 13.1 | 11.4 | 10.1 | 13.6 | -3.0 | 0.39 |  | 11.8 | 10.4 | 13.5 | 11.5 | 9.7 | 13.4 | -2.3 | 0.25 |  | 10.5 | 9.1 | 13.0 | 11.4 | 9.2 | 13.8 | 8.6 | 0.31 |
| Soluble dietary fibre | g/d | 2.8 | 2.5 | 3.1 | 2.8 | 2.4 | 3.5 | 0.0 | 0.33 |  | 2.9 | 2.5 | 3.4 | 2.8 | 2.3 | 3.4 | -5.2 | 0.22 |  | 2.5 | 2.2 | 3.1 | 2.8 | 2.1 | 3.4 | 13.3 | 0.23 |
| Insoluble dietary fibre | g/d | 8.5 | 7.5 | 9.3 | 8.4 | 7.5 | 9.7 | -1.6 | 0.46 |  | 8.5 | 7.5 | 9.5 | 8.3 | 7.2 | 9.6 | -1.7 | 0.25 |  | 7.8 | 6.4 | 9.3 | 8.3 | 6.9 | 10.1 | 5.8 | 0.31 |
| Retinol | μg/d | 258 | 212 | 292 | 355 | 296 | 550 | 37.6 | 0.32 |  | 248 | 205 | 300 | 345 | 259 | 529 | 38.9 | 0.28 |  | 216 | 176 | 287 | 302 | 220 | 435 | 39.7 | 0.31 |
| Vitamin A (retinol equivalent)^†^ | μg/d | 506 | 445 | 596 | 644 | 527 | 872 | 27.2 | 0.37 |  | 513 | 449 | 618 | 614 | 487 | 906 | 19.5 | 0.26 |  | 434 | 352 | 600 | 577 | 429 | 802 | 33.1 | 0.41 |
| α-carotene | μg/d | 616 | 456 | 805 | 350 | 280 | 548 | -43.3 | 0.27 |  | 642 | 476 | 900 | 340 | 230 | 594 | -47.0 | 0.17 |  | 462 | 274 | 798 | 330 | 149 | 601 | -28.5 | 0.22 |
| β-carotene | μg/d | 2728 | 2015 | 3188 | 2586 | 1973 | 3384 | -5.2 | 0.36 |  | 2739 | 1870 | 3450 | 2598 | 1766 | 3758 | -5.2 | 0.28 |  | 2222 | 1323 | 3244 | 2853 | 1533 | 3971 | 28.4 | 0.41 |
| β-carotene equivalent^‡^ | μg/d | 3181 | 2365 | 3772 | 2993 | 2309 | 3906 | -5.9 | 0.37 |  | 3178 | 2244 | 4060 | 2949 | 2047 | 4279 | -7.2 | 0.27 |  | 2588 | 1672 | 3723 | 3183 | 1753 | 4509 | 23.0 | 0.41 |
| Cryptoxanthin | μg/d | 148 | 74 | 332 | 292 | 196 | 394 | 97.3 | 0.39 |  | 90 | 41 | 345 | 211 | 97 | 378 | 134.8 | 0.35 |  | 64 | 20 | 295 | 200 | 66 | 458 | 209.9 | 0.28 |
| Vitamin D | μg/d | 7.3 | 5.4 | 8.6 | 11.6 | 9.2 | 15.2 | 59.5 | 0.22 |  | 6.5 | 4.6 | 8.8 | 10.8 | 7.3 | 14.4 | 65.6 | 0.19 |  | 6.8 | 4.8 | 9.3 | 8.4 | 5.7 | 14.2 | 24.4 | 0.10 |
| α-tocopherol | mg/d | 8.9 | 7.9 | 9.5 | 8.0 | 7.2 | 8.9 | -9.4 | 0.25 |  | 8.8 | 8.0 | 9.9 | 8.0 | 7.0 | 9.0 | -9.5 | 0.34 |  | 9.5 | 7.7 | 11.1 | 8.3 | 6.9 | 9.7 | -12.8 | 0.29 |
| Vitamin K | μg/d | 182 | 154 | 220 | 239 | 194 | 297 | 31.2 | 0.31 |  | 181 | 139 | 230 | 238 | 165 | 326 | 31.4 | 0.37 |  | 174 | 138 | 250 | 243 | 173 | 355 | 39.7 | 0.51 |
| Thiamine | mg/d | 1.1 | 1.0 | 1.2 | 0.9 | 0.8 | 1.0 | -21.2 | 0.13 |  | 1.1 | 1.0 | 1.3 | 0.9 | 0.8 | 1.0 | -22.5 | 0.08 |  | 1.1 | 1.0 | 1.3 | 0.9 | 0.7 | 1.0 | -24.6 | 0.30 |
| Riboflavin | mg/d | 1.4 | 1.2 | 1.5 | 1.5 | 1.3 | 1.7 | 12.6 | 0.41 |  | 1.3 | 1.2 | 1.5 | 1.5 | 1.3 | 1.8 | 15.9 | 0.38 |  | 1.2 | 1.1 | 1.5 | 1.5 | 1.2 | 1.8 | 17.7 | 0.50 |
| Niacin | mg/d | 16.1 | 14.6 | 17.4 | 15.2 | 13.5 | 17.3 | -5.5 | 0.18 |  | 16.0 | 14.2 | 18.2 | 14.5 | 12.5 | 16.3 | -9.1 | 0.04 |  | 15.9 | 13.5 | 18.9 | 14.6 | 12.4 | 18.0 | -7.8 | 0.32 |
| Vitamin B_6_ | mg/d | 1.3 | 1.2 | 1.4 | 1.2 | 1.1 | 1.4 | -3.1 | 0.29 |  | 1.3 | 1.1 | 1.5 | 1.2 | 1.1 | 1.4 | -8.5 | 0.09 |  | 1.2 | 1.0 | 1.5 | 1.2 | 1.0 | 1.4 | -2.4 | 0.43 |
| Vitamin B_12_ | μg/d | 5.5 | 4.1 | 6.7 | 7.5 | 6.0 | 9.4 | 37.9 | 0.32 |  | 5.2 | 3.8 | 6.7 | 6.9 | 5.2 | 9.4 | 33.6 | 0.34 |  | 4.6 | 3.1 | 6.3 | 6.1 | 4.5 | 8.7 | 33.3 | 0.29 |
| Folate | μg/d | 274 | 246 | 300 | 320 | 274 | 380 | 16.9 | 0.36 |  | 269 | 234 | 319 | 327 | 255 | 389 | 21.9 | 0.30 |  | 261 | 217 | 331 | 345 | 278 | 406 | 32.1 | 0.41 |
| Pantothenic acid | mg/d | 6.4 | 6.0 | 6.9 | 7.3 | 6.9 | 8.0 | 13.4 | 0.46 |  | 6.4 | 5.9 | 6.9 | 7.3 | 6.7 | 8.3 | 14.5 | 0.44 |  | 6.1 | 5.3 | 6.9 | 7.1 | 6.3 | 8.3 | 17.7 | 0.55 |
| Vitamin C | mg/d | 87.8 | 71.4 | 103.8 | 107.2 | 90.8 | 126.7 | 22.1 | 0.41 |  | 87.4 | 67.1 | 107.0 | 102.5 | 77.0 | 135.7 | 17.3 | 0.31 |  | 82.2 | 59.3 | 107.8 | 115.2 | 74.0 | 143.4 | 40.1 | 0.42 |
| Sodium | mg/d | 3845 | 3499 | 4154 | 3739 | 3315 | 4098 | -2.8 | 0.24 |  | 3922 | 3514 | 4320 | 3723 | 3230 | 4264 | -5.1 | 0.12 |  | 3832 | 3312 | 4384 | 3780 | 3314 | 4438 | -1.4 | 0.34 |
| Potassium | mg/d | 2481 | 2275 | 2664 | 2507 | 2266 | 2778 | 1.0 | 0.40 |  | 2479 | 2208 | 2792 | 2458 | 2154 | 2906 | -0.9 | 0.32 |  | 2285 | 1940 | 2698 | 2446 | 2034 | 2876 | 7.0 | 0.53 |
| Calcium | mg/d | 633 | 555 | 707 | 698 | 612 | 788 | 10.1 | 0.52 |  | 631 | 517 | 754 | 721 | 568 | 901 | 14.3 | 0.51 |  | 467 | 374 | 616 | 584 | 455 | 776 | 25.2 | 0.57 |
| Magnesium | mg/d | 242 | 225 | 267 | 255 | 238 | 282 | 5.4 | 0.43 |  | 243 | 224 | 269 | 255 | 225 | 283 | 5.0 | 0.27 |  | 227 | 201 | 263 | 247 | 206 | 283 | 8.7 | 0.54 |
| Phosphorus | mg/d | 1162 | 1083 | 1251 | 1192 | 1104 | 1296 | 2.5 | 0.49 |  | 1158 | 1057 | 1257 | 1186 | 1072 | 1402 | 2.4 | 0.42 |  | 1071 | 965 | 1211 | 1136 | 973 | 1329 | 6.0 | 0.60 |
| Iron | mg/d | 7.0 | 6.4 | 7.6 | 7.8 | 7.1 | 8.7 | 12.7 | 0.32 |  | 7.0 | 6.4 | 7.9 | 7.8 | 6.7 | 8.9 | 11.6 | 0.20 |  | 7.0 | 6.2 | 7.9 | 8.4 | 6.9 | 9.5 | 19.3 | 0.44 |
| Zinc | mg/d | 9.6 | 9.1 | 10.1 | 9.5 | 9.0 | 10.1 | -1.6 | 0.35 |  | 9.7 | 9.0 | 10.4 | 9.6 | 8.8 | 10.5 | -1.5 | 0.23 |  | 9.5 | 8.5 | 10.6 | 9.5 | 8.6 | 10.2 | -0.4 | 0.44 |
| Copper | mg/d | 1.1 | 1.0 | 1.2 | 1.3 | 1.2 | 1.4 | 13.6 | 0.46 |  | 1.1 | 1.0 | 1.2 | 1.2 | 1.1 | 1.4 | 12.7 | 0.34 |  | 1.1 | 1.0 | 1.2 | 1.3 | 1.1 | 1.4 | 15.5 | 0.57 |
| Manganese | mg/d | 2.9 | 2.7 | 3.2 | 3.7 | 3.1 | 4.3 | 27.3 | 0.26 |  | 2.9 | 2.6 | 3.2 | 3.8 | 3.0 | 4.4 | 31.3 | 0.22 |  | 3.1 | 2.7 | 3.8 | 3.9 | 3.1 | 4.7 | 24.6 | 0.36 |
|  |  |  |  |  |  |  |  |  |  |  |  |  |  |  |  |  |  |  |  |  |  |  |  |  |  |  |  |
| Median CC |  |  |  |  |  |  |  |  | 0.32 |  |  |  |  |  |  |  |  | 0.25 |  |  |  |  |  |  |  |  | 0.38 |

SFA, saturated fatty acids; MUFA, monounsaturated fatty acids; PUFA, polyunsaturated fatty acids; EPA, eicosapentaenoic acid; DPA, docosapentaenoic acid; DHA, docosahexaenoic acid; P25, 25th percentile; P75, 75th percentile.

^*^ Sum of EPA, n-3 DPA and DHA.

^†^ Sum of retinol, β-carotene/12, α-carotene/24 and cryptoxanthin/24.

^‡^ Sum of β-carotene, α-carotene/2 and cryptoxanthin/2.

^§^ Percentage differences: (BDHQ15y – 8-day DR)/8-day DR × 100 (%).

**Table ST4.** Median estimates of daily energy and energy-adjusted nutrient intakes using the density method from the 8-day weighed dietary record (DR) and the brief-type diet history questionnaire for Japanese adolescents and children (BDHQ15y) in 412 Japanese girls aged 6–17 years: percentage differences in median intakes and Spearman’s rank correlations coefficients (CCs), according to the age group.

|  |  | 6–9 years (*n* 144) | | | | | | | |  | 10–14 years (*n* 162) | | | | | | | |  | 15–17 years (*n* 106) | | | | | | | |
| --- | --- | --- | --- | --- | --- | --- | --- | --- | --- | --- | --- | --- | --- | --- | --- | --- | --- | --- | --- | --- | --- | --- | --- | --- | --- | --- | --- |
|  | Unit | 8-day DR | | | BDHQ15y | | | Median difference (%) ^§^ | CC |  | 8-day DR | | | BDHQ15y | | | Median difference (%) ^§^ | CC |  | 8-day DR | | | BDHQ15y | | | Median difference (%) ^§^ | CC |
|  |  | Median | P25 | P75 | Median | P25 | P75 |  |  |  | Median | P25 | P75 | Median | P25 | P75 |  |  |  | Median | P25 | P75 | Median | P25 | P75 |  |  |
| Protein | % energy | 14.3 | 13.1 | 15.1 | 13.9 | 12.8 | 15.1 | -3.1 | 0.38 |  | 14.4 | 13.4 | 15.2 | 14.3 | 12.7 | 15.5 | -0.5 | 0.33 |  | 14.3 | 13.1 | 15.5 | 14.2 | 12.9 | 15.6 | -0.8 | 0.39 |
| Fat | % energy | 31.0 | 28.7 | 33.2 | 30.5 | 27.1 | 33.1 | -1.5 | 0.20 |  | 32.0 | 29.6 | 34.0 | 30.7 | 28.5 | 33.7 | -4.2 | 0.25 |  | 32.8 | 30.1 | 35.1 | 30.7 | 26.7 | 33.6 | -6.4 | 0.10 |
| SFA | % energy | 10.4 | 9.3 | 11.4 | 9.6 | 8.3 | 11.2 | -7.6 | 0.30 |  | 10.3 | 9.5 | 11.5 | 9.6 | 8.3 | 11.1 | -7.3 | 0.25 |  | 10.1 | 9.0 | 10.9 | 9.2 | 7.7 | 10.3 | -8.8 | 0.26 |
| MUFA | % energy | 11.2 | 10.1 | 12.3 | 10.4 | 9.2 | 11.4 | -6.8 | 0.23 |  | 12.0 | 10.7 | 13.0 | 10.7 | 9.5 | 11.7 | -10.6 | 0.22 |  | 12.7 | 11.4 | 13.6 | 10.8 | 9.4 | 11.9 | -14.8 | 0.08 |
| PUFA | % energy | 5.7 | 5.1 | 6.4 | 6.2 | 5.5 | 7.0 | 8.4 | 0.23 |  | 5.9 | 5.2 | 6.4 | 6.5 | 5.8 | 7.2 | 10.8 | 0.13 |  | 6.1 | 5.6 | 6.9 | 6.6 | 5.7 | 7.6 | 7.8 | 0.13 |
| n-6 PUFA | % energy | 4.5 | 3.9 | 4.9 | 5.3 | 4.5 | 6.0 | 17.6 | 0.22 |  | 4.5 | 4.0 | 5.0 | 5.5 | 4.8 | 6.1 | 23.2 | 0.10 |  | 4.8 | 4.3 | 5.3 | 5.5 | 4.9 | 6.3 | 16.2 | 0.21 |
| n-3 PUFA | % energy | 0.88 | 0.72 | 1.05 | 1.07 | 0.93 | 1.28 | 21.6 | 0.23 |  | 0.86 | 0.70 | 1.04 | 1.13 | 0.96 | 1.25 | 31.4 | 0.14 |  | 0.91 | 0.75 | 1.10 | 1.16 | 0.97 | 1.34 | 27.5 | 0.18 |
| Marine-origin n-3 PUFA^*^ | % energy | 0.22 | 0.13 | 0.34 | 0.28 | 0.21 | 0.39 | 27.3 | 0.28 |  | 0.21 | 0.12 | 0.32 | 0.29 | 0.20 | 0.38 | 38.1 | 0.22 |  | 0.18 | 0.11 | 0.32 | 0.28 | 0.20 | 0.39 | 55.6 | 0.35 |
| EPA | % energy | 0.07 | 0.04 | 0.10 | 0.09 | 0.06 | 0.13 | 26.6 | 0.30 |  | 0.06 | 0.03 | 0.10 | 0.09 | 0.06 | 0.12 | 42.6 | 0.22 |  | 0.05 | 0.03 | 0.09 | 0.09 | 0.06 | 0.13 | 70.4 | 0.34 |
| n-3 DPA | % energy | 0.02 | 0.02 | 0.04 | 0.03 | 0.02 | 0.04 | 32.2 | 0.31 |  | 0.02 | 0.02 | 0.03 | 0.03 | 0.02 | 0.04 | 21.8 | 0.19 |  | 0.02 | 0.02 | 0.03 | 0.03 | 0.02 | 0.04 | 29.2 | 0.38 |
| DHA | % energy | 0.13 | 0.07 | 0.20 | 0.16 | 0.12 | 0.22 | 23.1 | 0.27 |  | 0.12 | 0.07 | 0.19 | 0.17 | 0.12 | 0.22 | 41.7 | 0.22 |  | 0.11 | 0.07 | 0.19 | 0.17 | 0.13 | 0.22 | 54.5 | 0.34 |
| α-linolenic acid | % energy | 0.60 | 0.52 | 0.71 | 0.76 | 0.64 | 0.88 | 26.7 | 0.20 |  | 0.61 | 0.51 | 0.69 | 0.78 | 0.68 | 0.90 | 27.9 | 0.19 |  | 0.66 | 0.57 | 0.77 | 0.81 | 0.68 | 0.96 | 22.7 | 0.24 |
| Cholesterol | mg/1000 kcal | 153 | 130 | 185 | 173 | 138 | 209 | 12.7 | 0.14 |  | 172 | 144 | 198 | 182 | 152 | 225 | 6.2 | 0.34 |  | 185 | 154 | 226 | 211 | 166 | 251 | 14.2 | 0.39 |
| Carbohydrate | % energy | 54.6 | 52.1 | 57.6 | 56.0 | 52.4 | 59.2 | 2.5 | 0.16 |  | 53.5 | 51.3 | 55.9 | 55.0 | 51.8 | 58.1 | 2.8 | 0.24 |  | 53.4 | 49.7 | 55.4 | 54.7 | 51.9 | 58.5 | 2.4 | 0.12 |
| Added sugar | % energy | 6.5 | 5.0 | 8.6 | 7.2 | 5.1 | 9.2 | 10.1 | 0.55 |  | 6.2 | 4.6 | 8.3 | 7.3 | 5.6 | 9.0 | 16.7 | 0.42 |  | 6.7 | 4.9 | 8.2 | 7.7 | 5.4 | 9.2 | 14.6 | 0.32 |
| Total dietary fibre | g/1000 kcal | 5.8 | 5.1 | 6.6 | 5.7 | 5.0 | 6.6 | -1.7 | 0.45 |  | 5.7 | 5.2 | 6.3 | 5.7 | 5.1 | 6.7 | 0.4 | 0.18 |  | 5.4 | 4.9 | 6.4 | 5.7 | 5.0 | 6.8 | 5.3 | 0.38 |
| Soluble dietary fibre | g/1000 kcal | 1.4 | 1.3 | 1.6 | 1.4 | 1.2 | 1.7 | -0.7 | 0.29 |  | 1.4 | 1.2 | 1.6 | 1.5 | 1.2 | 1.7 | 2.8 | 0.19 |  | 1.3 | 1.2 | 1.5 | 1.4 | 1.2 | 1.8 | 6.7 | 0.30 |
| Insoluble dietary fibre | g/1000 kcal | 4.1 | 3.7 | 4.7 | 4.2 | 3.6 | 4.7 | 1.0 | 0.44 |  | 4.1 | 3.7 | 4.5 | 4.2 | 3.7 | 4.8 | 1.2 | 0.25 |  | 3.8 | 3.4 | 4.6 | 4.2 | 3.6 | 4.9 | 9.4 | 0.41 |
| Retinol | μg/1000 kcal | 120 | 100 | 143 | 154 | 127 | 192 | 28.5 | 0.16 |  | 117 | 101 | 142 | 164 | 133 | 236 | 40.1 | 0.22 |  | 112 | 90 | 132 | 144 | 111 | 178 | 27.9 | 0.25 |
| Vitamin A (retinol equivalent)^†^ | μg/1000 kcal | 259 | 222 | 301 | 301 | 243 | 410 | 16.1 | 0.21 |  | 252 | 213 | 297 | 328 | 249 | 425 | 29.9 | 0.24 |  | 231 | 191 | 277 | 278 | 226 | 369 | 20.3 | 0.26 |
| α-carotene | μg/1000 kcal | 322 | 247 | 452 | 209 | 132 | 291 | -35.2 | 0.21 |  | 309 | 213 | 419 | 201 | 125 | 316 | -35.1 | 0.31 |  | 247 | 161 | 300 | 162 | 73 | 286 | -34.5 | 0.33 |
| β-carotene | μg/1000 kcal | 1329 | 1065 | 1735 | 1439 | 932 | 2023 | 8.3 | 0.32 |  | 1318 | 953 | 1685 | 1415 | 1003 | 1987 | 7.4 | 0.24 |  | 1120 | 902 | 1503 | 1339 | 874 | 2089 | 19.6 | 0.36 |
| β-carotene equivalent^‡^ | μg/1000 kcal | 1589 | 1316 | 2023 | 1630 | 1118 | 2262 | 2.5 | 0.31 |  | 1524 | 1163 | 1977 | 1579 | 1164 | 2256 | 3.6 | 0.26 |  | 1317 | 1058 | 1731 | 1518 | 1007 | 2263 | 15.3 | 0.36 |
| Cryptoxanthin | μg/1000 kcal | 116 | 32 | 213 | 150 | 84 | 248 | 30.1 | 0.43 |  | 80 | 25 | 147 | 138 | 77 | 232 | 72.9 | 0.40 |  | 39 | 22 | 153 | 114 | 57 | 234 | 191.1 | 0.49 |
| Vitamin D | μg/1000 kcal | 3.1 | 2.3 | 4.2 | 4.7 | 3.5 | 6.2 | 51.0 | 0.31 |  | 3.3 | 2.5 | 4.4 | 5.1 | 3.8 | 6.6 | 56.6 | 0.18 |  | 3.2 | 2.6 | 4.5 | 4.8 | 3.3 | 6.8 | 50.8 | 0.26 |
| α-tocopherol | mg/1000 kcal | 4.2 | 3.7 | 4.7 | 4.0 | 3.5 | 4.5 | -5.5 | 0.16 |  | 4.3 | 3.9 | 5.0 | 4.1 | 3.6 | 4.6 | -4.5 | 0.07 |  | 4.7 | 4.2 | 5.2 | 4.0 | 3.6 | 4.7 | -14.3 | -0.04 |
| Vitamin K | μg/1000 kcal | 89 | 69 | 111 | 118 | 84 | 158 | 32.5 | 0.47 |  | 90 | 75 | 113 | 116 | 91 | 156 | 29.9 | 0.34 |  | 98 | 78 | 125 | 124 | 95 | 174 | 26.9 | 0.37 |
| Thiamine | mg/1000 kcal | 0.5 | 0.5 | 0.6 | 0.4 | 0.4 | 0.5 | -21.6 | 0.36 |  | 0.5 | 0.5 | 0.6 | 0.4 | 0.4 | 0.5 | -21.2 | 0.17 |  | 0.5 | 0.4 | 0.6 | 0.4 | 0.4 | 0.5 | -17.6 | 0.22 |
| Riboflavin | mg/1000 kcal | 0.6 | 0.6 | 0.7 | 0.7 | 0.7 | 0.8 | 10.9 | 0.24 |  | 0.6 | 0.6 | 0.7 | 0.7 | 0.7 | 0.8 | 17.7 | 0.26 |  | 0.6 | 0.5 | 0.7 | 0.7 | 0.6 | 0.8 | 20.7 | 0.38 |
| Niacin | mg/1000 kcal | 7.1 | 6.2 | 8.0 | 7.0 | 6.1 | 8.2 | -2.4 | 0.31 |  | 7.4 | 6.6 | 8.5 | 7.2 | 6.2 | 8.3 | -2.8 | 0.26 |  | 7.7 | 6.7 | 9.0 | 7.5 | 6.8 | 8.9 | -2.6 | 0.30 |
| Vitamin B_6_ | mg/1000 kcal | 0.6 | 0.5 | 0.6 | 0.6 | 0.5 | 0.7 | -5.0 | 0.41 |  | 0.6 | 0.6 | 0.7 | 0.6 | 0.5 | 0.7 | -4.9 | 0.22 |  | 0.6 | 0.5 | 0.7 | 0.6 | 0.5 | 0.7 | 5.2 | 0.42 |
| Vitamin B_12_ | μg/1000 kcal | 2.4 | 1.8 | 3.3 | 3.2 | 2.6 | 4.3 | 33.1 | 0.23 |  | 2.3 | 1.8 | 3.1 | 3.4 | 2.6 | 4.3 | 48.5 | 0.22 |  | 2.1 | 1.7 | 2.9 | 3.3 | 2.6 | 4.4 | 62.1 | 0.21 |
| Folate | μg/1000 kcal | 135 | 117 | 153 | 160 | 132 | 195 | 18.5 | 0.26 |  | 136 | 122 | 155 | 161.3 | 137 | 198.7 | 18.4 | 0.24 |  | 137 | 120 | 159 | 165 | 142 | 206 | 20.5 | 0.29 |
| Pantothenic acid | mg/1000 kcal | 3.1 | 2.9 | 3.3 | 3.4 | 3.1 | 3.7 | 10.8 | 0.38 |  | 3.0 | 2.8 | 3.2 | 3.5 | 3.2 | 3.8 | 16.4 | 0.31 |  | 2.8 | 2.5 | 3.0 | 3.3 | 3.0 | 3.7 | 19.6 | 0.48 |
| Vitamin C | mg/1000 kcal | 43.3 | 36.0 | 55.1 | 55.5 | 44.1 | 68.6 | 28.1 | 0.30 |  | 44.0 | 35.5 | 54.1 | 56.6 | 43.7 | 69.9 | 28.7 | 0.33 |  | 41.7 | 33.2 | 54.1 | 55.6 | 44.9 | 75.7 | 33.2 | 0.45 |
| Sodium | mg/1000 kcal | 1843 | 1726 | 2059 | 1826 | 1600 | 2061 | -0.9 | 0.20 |  | 1814 | 1657 | 2028 | 1845 | 1669 | 2008 | 1.7 | 0.21 |  | 1895 | 1639 | 2082 | 1840 | 1616 | 2035 | -2.9 | 0.30 |
| Potassium | mg/1000 kcal | 1206 | 1100 | 1296 | 1202 | 1063 | 1396 | -0.3 | 0.43 |  | 1178 | 1079 | 1268 | 1206 | 1065 | 1409 | 2.4 | 0.31 |  | 1069 | 983 | 1240 | 1141 | 1002 | 1335 | 6.7 | 0.42 |
| Calcium | mg/1000 kcal | 310 | 273 | 349 | 318 | 282 | 387 | 2.7 | 0.35 |  | 278 | 236 | 333 | 314 | 265 | 365 | 13.2 | 0.40 |  | 232 | 194 | 272 | 276 | 227 | 338 | 19.3 | 0.45 |
| Magnesium | mg/1000 kcal | 118 | 107 | 130 | 119 | 107 | 132 | 0.4 | 0.49 |  | 115 | 106 | 123 | 120 | 108 | 133 | 4.5 | 0.23 |  | 110 | 99 | 123 | 120 | 105 | 137 | 9.2 | 0.48 |
| Phosphorus | mg/1000 kcal | 546 | 510 | 581 | 559 | 508 | 613 | 2.5 | 0.30 |  | 531 | 489 | 572 | 562 | 502 | 614 | 5.9 | 0.40 |  | 501 | 464 | 553 | 541 | 477 | 615 | 7.9 | 0.41 |
| Iron | mg/1000 kcal | 3.4 | 3.0 | 3.7 | 3.7 | 3.3 | 4.3 | 7.9 | 0.31 |  | 3.5 | 3.2 | 3.7 | 3.9 | 3.5 | 4.4 | 12.5 | 0.14 |  | 3.5 | 3.1 | 3.8 | 4.1 | 3.7 | 4.6 | 17.1 | 0.36 |
| Zinc | mg/1000 kcal | 4.3 | 4.0 | 4.6 | 4.3 | 4.0 | 4.7 | -0.7 | 0.36 |  | 4.3 | 4.1 | 4.7 | 4.4 | 4.0 | 4.7 | 0.9 | 0.22 |  | 4.4 | 4.1 | 4.8 | 4.4 | 4.0 | 4.8 | 0.7 | 0.37 |
| Copper | mg/1000 kcal | 0.5 | 0.5 | 0.6 | 0.6 | 0.5 | 0.6 | 13.7 | 0.49 |  | 0.5 | 0.5 | 0.6 | 0.6 | 0.5 | 0.6 | 13.7 | 0.31 |  | 0.5 | 0.5 | 0.6 | 0.6 | 0.6 | 0.7 | 15.4 | 0.43 |
| Manganese | mg/1000 kcal | 1.3 | 1.2 | 1.5 | 1.8 | 1.4 | 2.1 | 33.3 | 0.12 |  | 1.4 | 1.2 | 1.7 | 1.8 | 1.5 | 2.1 | 30.4 | 0.24 |  | 1.5 | 1.3 | 1.7 | 1.9 | 1.5 | 2.3 | 29.9 | 0.21 |
|  |  |  |  |  |  |  |  |  |  |  |  |  |  |  |  |  |  |  |  |  |  |  |  |  |  |  |  |
| Median |  |  |  |  |  |  |  |  | 0.30 |  |  |  |  |  |  |  |  | 0.24 |  |  |  |  |  |  |  |  | 0.34 |

SFA, saturated fatty acids; MUFA, monounsaturated fatty acids; PUFA, polyunsaturated fatty acids; EPA, eicosapentaenoic acid; DPA, docosapentaenoic acid; DHA, docosahexaenoic acid; P25, 25th percentile; P75, 75th percentile.

^*^ Sum of EPA, n-3 DPA and DHA.

^†^ Sum of retinol, β-carotene/12, α-carotene/24 and cryptoxanthin/24.

^‡^ Sum of β-carotene, α-carotene/2 and cryptoxanthin/2.

^§^ Percentage differences: (BDHQ15y – 8-day DR)/8-day DR × 100 (%).

**Table ST5.** Median estimates of daily energy and energy-adjusted nutrient intakes using the residual method from the 8-day weighed dietary record (DR) and the brief-type diet history questionnaire for Japanese adolescents and children (BDHQ15y) in 412 Japanese girls aged 6–17 years: percentage differences in median intakes and Spearman’s rank correlations coefficients (CCs), according to the age group.

|  |  | 6–9 years (*n* 144) | | | | | | | |  | 10–14 years (*n* 162) | | | | | | | |  | 15–17 years (*n* 106) | | | | | | | |
| --- | --- | --- | --- | --- | --- | --- | --- | --- | --- | --- | --- | --- | --- | --- | --- | --- | --- | --- | --- | --- | --- | --- | --- | --- | --- | --- | --- |
|  | Unit | 8-day DR | | | BDHQ15y | | | Median difference (%) ^§^ | CC |  | 8-day DR | | | BDHQ15y | | | Median difference (%) ^§^ | CC |  | 8-day DR | | | BDHQ15y | | | Median difference (%^) §^ | CC |
|  |  | Median | P25 | P75 | Median | P25 | P75 |  |  |  | Median | P25 | P75 | Median | P25 | P75 |  |  |  | Median | P25 | P75 | Median | P25 | P75 |  |  |
| Energy | kcal/d | 1708 | 1533 | 1877 | 1684 | 1359 | 1941 | -1.4 | 0.32 |  | 2030 | 1840 | 2235 | 2087 | 1662 | 2423 | 2.8 | 0.26 |  | 1956 | 1690 | 2261 | 2016 | 1741 | 2307 | 3.1 | 0.29 |
| Protein | g/d | 77.7 | 72.3 | 80.4 | 73.7 | 69.7 | 79.0 | -5.1 | 0.36 |  | 78.8 | 74.0 | 82.7 | 77.0 | 69.3 | 83.2 | -2.3 | 0.31 |  | 77.8 | 72.4 | 83.7 | 77.2 | 70.5 | 83.3 | -0.9 | 0.36 |
| Fat | g/d | 75.2 | 71.2 | 79.8 | 70.0 | 64.6 | 74.8 | -6.9 | 0.19 |  | 77.9 | 72.6 | 82.8 | 72.9 | 67.6 | 81.1 | -6.4 | 0.19 |  | 79.0 | 73.5 | 84.4 | 73.3 | 64.2 | 82.0 | -7.3 | 0.08 |
| SFA | g/d | 24.6 | 22.7 | 26.7 | 22.4 | 20.0 | 25.4 | -9.0 | 0.33 |  | 25.1 | 23.2 | 27.7 | 22.7 | 20.1 | 26.6 | -9.5 | 0.24 |  | 24.4 | 22.0 | 26.4 | 21.8 | 18.7 | 25.6 | -10.7 | 0.23 |
| MUFA | g/d | 28.0 | 26.2 | 30.1 | 23.8 | 21.7 | 25.8 | -14.9 | 0.20 |  | 29.3 | 26.6 | 31.6 | 25.3 | 22.8 | 28.4 | -13.7 | 0.17 |  | 30.8 | 28.4 | 33.4 | 25.5 | 22.3 | 29.5 | -17.1 | 0.07 |
| PUFA | g/d | 13.9 | 12.6 | 15.0 | 14.1 | 12.9 | 15.7 | 1.9 | 0.26 |  | 14.2 | 12.8 | 15.5 | 15.4 | 13.8 | 17.4 | 8.5 | 0.09 |  | 14.7 | 13.5 | 16.2 | 15.6 | 13.8 | 18.0 | 6.3 | 0.05 |
| n-6 PUFA | g/d | 10.9 | 9.7 | 11.8 | 12.0 | 10.8 | 13.3 | 10.0 | 0.25 |  | 10.9 | 9.8 | 12.2 | 13.0 | 11.5 | 14.6 | 19.2 | 0.04 |  | 11.5 | 10.6 | 12.8 | 13.3 | 11.6 | 15.1 | 15.6 | 0.16 |
| n-3 PUFA | g/d | 2.02 | 1.75 | 2.39 | 2.48 | 2.21 | 2.83 | 22.8 | 0.24 |  | 2.09 | 1.71 | 2.54 | 2.67 | 2.31 | 3.04 | 27.8 | 0.13 |  | 2.21 | 1.82 | 2.62 | 2.77 | 2.36 | 3.20 | 25.3 | 0.12 |
| Marine-origin n-3 PUFA^*^ | g/d | 0.48 | 0.31 | 0.71 | 0.66 | 0.53 | 0.85 | 37.5 | 0.28 |  | 0.50 | 0.29 | 0.75 | 0.68 | 0.51 | 0.92 | 36.0 | 0.21 |  | 0.44 | 0.28 | 0.70 | 0.68 | 0.51 | 0.94 | 54.5 | 0.35 |
| EPA | g/d | 0.15 | 0.09 | 0.22 | 0.21 | 0.17 | 0.27 | 39.0 | 0.29 |  | 0.14 | 0.08 | 0.23 | 0.22 | 0.15 | 0.30 | 49.3 | 0.20 |  | 0.13 | 0.07 | 0.22 | 0.21 | 0.14 | 0.29 | 67.2 | 0.35 |
| n-3 DPA | g/d | 0.05 | 0.04 | 0.08 | 0.07 | 0.06 | 0.08 | 32.6 | 0.29 |  | 0.06 | 0.04 | 0.08 | 0.07 | 0.05 | 0.09 | 22.8 | 0.19 |  | 0.06 | 0.04 | 0.08 | 0.07 | 0.05 | 0.10 | 24.1 | 0.38 |
| DHA | g/d | 0.28 | 0.18 | 0.41 | 0.38 | 0.30 | 0.48 | 35.7 | 0.27 |  | 0.30 | 0.17 | 0.43 | 0.39 | 0.30 | 0.51 | 30.0 | 0.22 |  | 0.26 | 0.17 | 0.41 | 0.40 | 0.32 | 0.55 | 53.8 | 0.34 |
| α-linolenic acid | g/d | 1.45 | 1.31 | 1.66 | 1.71 | 1.52 | 1.95 | 17.9 | 0.22 |  | 1.48 | 1.24 | 1.69 | 1.84 | 1.62 | 2.11 | 24.3 | 0.14 |  | 1.56 | 1.39 | 1.81 | 1.91 | 1.65 | 2.29 | 22.4 | 0.18 |
| Cholesterol | mg/d | 338 | 298 | 396 | 360 | 309 | 427 | 6.4 | 0.14 |  | 372 | 319 | 425 | 401 | 333 | 488 | 7.5 | 0.31 |  | 391 | 339 | 479 | 465 | 374 | 505 | 18.9 | 0.33 |
| Carbohydrate | g/d | 296 | 285 | 308 | 306 | 294 | 322 | 3.5 | 0.18 |  | 289 | 275 | 300 | 295 | 279 | 312 | 2.3 | 0.19 |  | 288 | 268 | 299 | 297 | 274 | 312 | 3.1 | 0.04 |
| Added sugar | g/d | 35 | 28 | 44 | 42 | 34 | 50 | 20.3 | 0.47 |  | 34.0 | 26.5 | 43.9 | 41 | 32 | 49 | 20.4 | 0.35 |  | 35.6 | 28.5 | 44.6 | 43 | 33 | 50 | 19.6 | 0.25 |
| Total dietary fibre | g/d | 11.9 | 10.8 | 13.0 | 12.0 | 10.6 | 13.6 | 1.1 | 0.51 |  | 12.3 | 11.3 | 13.3 | 12.4 | 11.0 | 14.7 | 1.4 | 0.19 |  | 11.6 | 10.6 | 13.6 | 12.4 | 10.9 | 14.3 | 6.6 | 0.39 |
| Soluble dietary fibre | g/d | 2.9 | 2.6 | 3.2 | 2.9 | 2.5 | 3.4 | 1.0 | 0.39 |  | 3.0 | 2.7 | 3.3 | 3.1 | 2.6 | 3.8 | 4.3 | 0.21 |  | 2.8 | 2.5 | 3.3 | 3.1 | 2.6 | 3.7 | 7.4 | 0.32 |
| Insoluble dietary fibre | g/d | 8.4 | 7.7 | 9.3 | 8.8 | 7.7 | 9.7 | 4.5 | 0.47 |  | 8.9 | 8.0 | 9.7 | 8.9 | 8.0 | 10.5 | 0.2 | 0.26 |  | 8.2 | 7.3 | 9.9 | 9.0 | 7.9 | 10.4 | 8.9 | 0.41 |
| Retinol | μg/d | 254 | 218 | 290 | 363 | 314 | 419 | 43.0 | 0.13 |  | 253 | 222 | 301 | 364 | 304 | 504 | 44.0 | 0.19 |  | 242 | 201 | 292 | 317 | 260 | 394 | 31.0 | 0.17 |
| Vitamin A (retinol equivalent)^†^ | μg/d | 523 | 464 | 598 | 648 | 563 | 823 | 24.0 | 0.23 |  | 531 | 460 | 631 | 723 | 554 | 915 | 36.3 | 0.24 |  | 484 | 406 | 577 | 604 | 501 | 768 | 24.7 | 0.25 |
| α-carotene | μg/d | 610 | 482 | 798 | 363 | 285 | 568 | -40.4 | 0.23 |  | 658 | 473 | 872 | 418 | 274 | 648 | -36.5 | 0.29 |  | 500 | 339 | 657 | 339 | 179 | 608 | -32.2 | 0.33 |
| β-carotene | μg/d | 2672 | 2174 | 3228 | 2786 | 2088 | 3897 | 4.3 | 0.37 |  | 2806 | 2092 | 3462 | 3013 | 2189 | 4235 | 7.4 | 0.22 |  | 2400 | 1923 | 3143 | 2864 | 1914 | 4233 | 19.4 | 0.34 |
| β-carotene equivalent^‡^ | μg/d | 3149 | 2630 | 3844 | 3152 | 2474 | 4397 | 0.1 | 0.38 |  | 3292 | 2474 | 4070 | 3478 | 2541 | 4820 | 5.6 | 0.24 |  | 2781 | 2213 | 3669 | 3185 | 2236 | 4762 | 14.5 | 0.35 |
| Cryptoxanthin | μg/d | 233 | 95 | 390 | 341 | 245 | 521 | 46.2 | 0.45 |  | 175 | 70 | 314 | 306 | 198 | 506 | 75.2 | 0.38 |  | 98 | 63 | 347 | 252 | 162 | 478 | 156.3 | 0.48 |
| Vitamin D | μg/d | 6.6 | 5.0 | 8.4 | 11.5 | 9.4 | 13.7 | 75.2 | 0.34 |  | 7.1 | 5.4 | 9.4 | 11.7 | 8.6 | 14.3 | 65.4 | 0.17 |  | 6.7 | 5.7 | 9.2 | 11.1 | 7.9 | 14.7 | 64.2 | 0.30 |
| α-tocopherol | mg/d | 8.9 | 8.2 | 9.8 | 8.3 | 7.5 | 9.1 | -7.0 | 0.17 |  | 9.4 | 8.4 | 10.5 | 8.8 | 7.8 | 10.0 | -6.8 | 0.07 |  | 10.1 | 9.1 | 11.0 | 8.7 | 7.7 | 9.9 | -13.5 | -0.07 |
| Vitamin K | μg/d | 191 | 158 | 232 | 242 | 191 | 323 | 27.1 | 0.47 |  | 196 | 166 | 245 | 252 | 204 | 337 | 28.6 | 0.36 |  | 209 | 171 | 255 | 277 | 207 | 362 | 32.3 | 0.38 |
| Thiamine | mg/d | 1.1 | 1.0 | 1.2 | 0.9 | 0.8 | 1.0 | -21.6 | 0.35 |  | 1.1 | 1.0 | 1.2 | 0.9 | 0.8 | 1.0 | -21.1 | 0.17 |  | 1.1 | 1.0 | 1.2 | 0.9 | 0.8 | 1.0 | -19.6 | 0.19 |
| Riboflavin | mg/d | 1.3 | 1.2 | 1.5 | 1.5 | 1.4 | 1.6 | 12.8 | 0.32 |  | 1.4 | 1.2 | 1.5 | 1.6 | 1.4 | 1.7 | 16.3 | 0.26 |  | 1.3 | 1.2 | 1.4 | 1.5 | 1.3 | 1.7 | 21.6 | 0.36 |
| Niacin | mg/d | 15.9 | 14.4 | 17.4 | 14.7 | 13.5 | 16.9 | -7.4 | 0.32 |  | 16.4 | 14.8 | 18.5 | 15.4 | 13.5 | 17.8 | -5.7 | 0.26 |  | 17.0 | 14.9 | 18.9 | 16.3 | 14.7 | 19.0 | -4.5 | 0.29 |
| Vitamin B_6_ | mg/d | 1.3 | 1.2 | 1.4 | 1.2 | 1.1 | 1.4 | -7.7 | 0.40 |  | 1.3 | 1.2 | 1.4 | 1.3 | 1.1 | 1.5 | -3.8 | 0.22 |  | 1.3 | 1.2 | 1.4 | 1.3 | 1.1 | 1.5 | 2.3 | 0.41 |
| Vitamin B_12_ | μg/d | 5.0 | 4.0 | 6.5 | 7.0 | 6.1 | 8.9 | 39.3 | 0.21 |  | 5.0 | 3.9 | 6.7 | 7.5 | 5.9 | 9.2 | 48.4 | 0.22 |  | 4.5 | 3.7 | 6.4 | 7.3 | 5.6 | 9.6 | 62.8 | 0.20 |
| Folate | μg/d | 283 | 251 | 318 | 324 | 280 | 372 | 14.4 | 0.32 |  | 295 | 263 | 332 | 344 | 296 | 410 | 16.7 | 0.27 |  | 297 | 258 | 345 | 352 | 309 | 429 | 18.5 | 0.30 |
| Pantothenic acid | mg/d | 6.5 | 6.1 | 6.9 | 7.3 | 6.8 | 8.0 | 11.8 | 0.42 |  | 6.5 | 6.1 | 6.9 | 7.5 | 6.9 | 8.2 | 15.7 | 0.31 |  | 6.0 | 5.6 | 6.5 | 7.1 | 6.5 | 7.8 | 19.4 | 0.44 |
| Vitamin C | mg/d | 91.4 | 77.8 | 108.9 | 111.9 | 94.4 | 139.2 | 22.4 | 0.35 |  | 93.5 | 77.8 | 114.9 | 120.8 | 95.4 | 145.8 | 29.1 | 0.38 |  | 89.8 | 73.3 | 117.7 | 117.0 | 97.3 | 147.8 | 30.3 | 0.45 |
| Sodium | mg/d | 3844 | 3604 | 4151 | 3673 | 3385 | 4195 | -4.4 | 0.20 |  | 3865 | 3606 | 4320 | 3970 | 3542 | 4355 | 2.7 | 0.18 |  | 4062 | 3545 | 4456 | 3928 | 3539 | 4349 | -3.3 | 0.30 |
| Potassium | mg/d | 2502 | 2305 | 2695 | 2525 | 2293 | 2782 | 0.9 | 0.48 |  | 2559 | 2321 | 2722 | 2603 | 2288 | 2964 | 1.7 | 0.27 |  | 2331 | 2139 | 2640 | 2488 | 2187 | 2859 | 6.8 | 0.42 |
| Calcium | mg/d | 614 | 557 | 690 | 686 | 625 | 805 | 11.7 | 0.37 |  | 595 | 512 | 694 | 683 | 592 | 777 | 14.7 | 0.37 |  | 509 | 423 | 582 | 616 | 501 | 733 | 21.2 | 0.47 |
| Magnesium | mg/d | 247 | 229 | 266 | 256 | 234 | 276 | 3.6 | 0.51 |  | 249 | 231 | 263 | 261 | 237 | 286 | 4.9 | 0.22 |  | 238 | 215 | 264 | 261 | 230 | 291 | 9.8 | 0.50 |
| Phosphorus | mg/d | 1147 | 1091 | 1209 | 1192 | 1110 | 1284 | 3.9 | 0.33 |  | 1151 | 1075 | 1237 | 1213 | 1095 | 1328 | 5.3 | 0.41 |  | 1087 | 1022 | 1182 | 1183 | 1034 | 1316 | 8.8 | 0.43 |
| Iron | mg/d | 7.2 | 6.5 | 7.8 | 7.7 | 7.1 | 8.7 | 8.0 | 0.32 |  | 7.4 | 7.0 | 7.9 | 8.3 | 7.6 | 9.5 | 11.9 | 0.11 |  | 7.5 | 6.6 | 8.1 | 8.6 | 7.9 | 9.7 | 15.1 | 0.32 |
| Zinc | mg/d | 9.5 | 9.1 | 10.0 | 9.3 | 8.9 | 9.9 | -2.2 | 0.34 |  | 9.6 | 9.0 | 10.2 | 9.5 | 8.8 | 10.2 | -0.3 | 0.23 |  | 9.6 | 9.0 | 10.4 | 9.5 | 8.8 | 10.3 | -1.0 | 0.35 |
| Copper | mg/d | 1.1 | 1.0 | 1.2 | 1.2 | 1.2 | 1.3 | 11.7 | 0.47 |  | 1.1 | 1.0 | 1.2 | 1.3 | 1.2 | 1.4 | 12.5 | 0.32 |  | 1.1 | 1.1 | 1.2 | 1.3 | 1.2 | 1.4 | 15.0 | 0.43 |
| Manganese | mg/d | 3.0 | 2.7 | 3.2 | 3.7 | 3.1 | 4.2 | 24.0 | 0.13 |  | 3.1 | 2.7 | 3.6 | 3.9 | 3.2 | 4.3 | 26.6 | 0.27 |  | 3.2 | 2.9 | 3.6 | 4.1 | 3.3 | 4.8 | 26.5 | 0.24 |
|  |  |  |  |  |  |  |  |  |  |  |  |  |  |  |  |  |  |  |  |  |  |  |  |  |  |  |  |
| Median CC |  |  |  |  |  |  |  |  | 0.32 |  |  |  |  |  |  |  |  | 0.22 |  |  |  |  |  |  |  |  | 0.33 |

SFA, saturated fatty acids; MUFA, monounsaturated fatty acids; PUFA, polyunsaturated fatty acids; EPA, eicosapentaenoic acid; DPA, docosapentaenoic acid; DHA, docosahexaenoic acid; P25, 25th percentile; P75, 75th percentile.

^*^ Sum of EPA, n-3 DPA and DHA.

^†^ Sum of retinol, β-carotene/12, α-carotene/24 and cryptoxanthin/24.

^‡^ Sum of β-carotene, α-carotene/2 and cryptoxanthin/2.

^§^ Percentage differences: (BDHQ15y – 8-day DR)/8-day DR × 100 (%).

**Table ST6.** Median estimates of energy-adjusted food group intakes using the density method from the 8-day weighed dietary record (DR) and the brief-type diet history questionnaire for Japanese adolescents and children (BDHQ15y) and their correlations in 432 Japanese boys aged 6–17 years: percentage differences in median intakes and Spearman’s rank correlations coefficients (CCs), according to the age group.

| Food group (g/1000 kcal)) | 6–9 years (*n* 126) | | | | | | | |  | 10–14 years (*n* 184) | | | | | | | |  | 15–17 years (*n* 122) | | | | | | | |
| --- | --- | --- | --- | --- | --- | --- | --- | --- | --- | --- | --- | --- | --- | --- | --- | --- | --- | --- | --- | --- | --- | --- | --- | --- | --- | --- |
|  | 8-day DR | | | BDHQ15y | | | Median difference (%)^*^ | CC |  | 8-day DR | | | BDHQ15y | | | Median difference (%)^*^ | CC |  | 8-day DR | | | BDHQ15y | | | Median difference (%)^*^ | CC |
|  | Median | P25 | P75 | Median | P25 | P75 |  |  |  | Median | P25 | P75 | Median | P25 | P75 |  |  |  | Median | P25 | P75 | Median | P25 | P75 |  |  |
| Grains | 211 | 194 | 233 | 227 | 188 | 260 | 7.3 | 0.32 |  | 226 | 201 | 254 | 229 | 191 | 278 | 1.3 | 0.27 |  | 245 | 217 | 282 | 241 | 193 | 281 | -1.4 | 0.49 |
| Rice | 136 | 116 | 170 | 169 | 140 | 217 | 24.8 | 0.40 |  | 153 | 131 | 184 | 188 | 143 | 241 | 22.7 | 0.28 |  | 183 | 156 | 217 | 190 | 141 | 245 | 3.6 | 0.42 |
| Bread | 24.1 | 14.5 | 31.4 | 15.7 | 7.0 | 28.1 | -34.7 | 0.39 |  | 19.5 | 12.4 | 28.0 | 13.1 | 8.0 | 22.1 | -32.9 | 0.44 |  | 18.3 | 8.8 | 28.0 | 15.5 | 8.2 | 29.7 | -15.3 | 0.53 |
| Noodles | 32.9 | 20.2 | 48.4 | 26.2 | 21.3 | 36.0 | -20.4 | 0.15 |  | 36.3 | 24.2 | 48.1 | 23.8 | 18.5 | 33.1 | -34.5 | 0.28 |  | 32.2 | 18.3 | 45.9 | 22.7 | 15.8 | 37.0 | -29.6 | 0.20 |
| Potatoes | 22.4 | 15.7 | 30.4 | 16.6 | 9.9 | 23.5 | -25.6 | 0.14 |  | 21.5 | 14.6 | 28.1 | 13.9 | 8.9 | 20.8 | -35.3 | 0.35 |  | 15.2 | 9.9 | 23.1 | 14.3 | 9.1 | 20.3 | -6.1 | 0.28 |
| Pulses | 15.6 | 9.2 | 25.3 | 22.3 | 15.1 | 31.6 | 42.7 | 0.34 |  | 16.5 | 11.1 | 24.0 | 20.9 | 12.6 | 32.0 | 26.8 | 0.31 |  | 14.4 | 7.8 | 19.5 | 20.8 | 14.0 | 30.9 | 44.1 | 0.26 |
| Total vegetables | 103.1 | 83.1 | 124.6 | 98.0 | 72.4 | 134.0 | -4.9 | 0.48 |  | 97.6 | 74.0 | 122.7 | 88.3 | 62.4 | 121.1 | -9.5 | 0.30 |  | 88.7 | 70.5 | 111.6 | 89.1 | 64.5 | 120.0 | 0.4 | 0.47 |
| Green and yellow vegetables | 30.8 | 22.0 | 39.8 | 32.2 | 20.8 | 47.5 | 4.5 | 0.48 |  | 28.0 | 17.9 | 37.7 | 29.7 | 20.8 | 44.6 | 6.2 | 0.39 |  | 23.9 | 16.0 | 38.3 | 30.3 | 19.0 | 42.4 | 26.6 | 0.45 |
| Other vegetables | 61.8 | 50.4 | 77.2 | 54.5 | 37.3 | 68.6 | -11.7 | 0.35 |  | 60.4 | 43.1 | 74.2 | 47.6 | 33.2 | 68.4 | -21.2 | 0.24 |  | 55.2 | 44.0 | 66.4 | 49.4 | 34.3 | 61.1 | -10.4 | 0.41 |
| Pickled vegetables | 0.4 | 0.0 | 1.7 | 0.7 | 0.0 | 2.7 | 56.8 | 0.27 |  | 0.7 | 0.1 | 1.6 | 0.8 | 0.0 | 3.4 | 15.2 | 0.20 |  | 1.0 | 0.2 | 2.0 | 0.9 | 0.0 | 3.6 | -6.3 | 0.35 |
| Mushrooms | 4.0 | 2.5 | 6.6 | 4.5 | 2.2 | 7.9 | 11.7 | 0.42 |  | 3.7 | 1.9 | 6.0 | 3.4 | 1.5 | 6.0 | -8.0 | 0.38 |  | 2.7 | 1.1 | 5.1 | 3.9 | 1.7 | 5.9 | 45.9 | 0.37 |
| Seaweeds | 2.9 | 1.7 | 5.4 | 3.5 | 2.0 | 6.7 | 20.8 | 0.25 |  | 2.7 | 1.6 | 5.1 | 3.5 | 1.7 | 6.0 | 27.8 | 0.33 |  | 2.5 | 1.2 | 4.4 | 2.8 | 1.2 | 5.1 | 13.8 | 0.31 |
| Fruits | 25.3 | 12.2 | 41.4 | 27.5 | 17.0 | 50.2 | 8.6 | 0.60 |  | 18.8 | 7.6 | 40.2 | 22.6 | 10.2 | 39.7 | 20.3 | 0.46 |  | 13.7 | 4.7 | 26.4 | 16.9 | 8.4 | 36.0 | 23.2 | 0.43 |
| Fish and shellfish | 17.5 | 11.6 | 28.8 | 27.3 | 20.3 | 39.8 | 56.1 | 0.34 |  | 18.0 | 11.2 | 25.2 | 25.2 | 17.1 | 35.0 | 40.3 | 0.30 |  | 17.4 | 10.1 | 24.1 | 24.1 | 15.7 | 33.2 | 38.7 | 0.34 |
| Meat | 48.8 | 38.1 | 57.5 | 34.9 | 28.9 | 43.7 | -28.4 | 0.16 |  | 54.3 | 45.8 | 64.3 | 34.4 | 28.8 | 44.6 | -36.7 | 0.07 |  | 59.9 | 49.2 | 75.6 | 35.9 | 28.8 | 49.5 | -40.0 | 0.20 |
| Eggs | 18.7 | 12.0 | 28.3 | 15.3 | 9.7 | 24.3 | -18.2 | 0.43 |  | 17.8 | 13.9 | 25.5 | 17.6 | 11.1 | 24.6 | -1.0 | 0.41 |  | 22.9 | 16.8 | 29.2 | 21.5 | 14.7 | 26.9 | -6.4 | 0.40 |
| Dairy products | 122.0 | 80.0 | 164.8 | 97.4 | 67.2 | 149.4 | -20.2 | 0.49 |  | 101.3 | 68.6 | 141.0 | 106.9 | 66.0 | 169.7 | 5.5 | 0.60 |  | 51.5 | 24.2 | 89.7 | 64.1 | 28.0 | 139.5 | 24.6 | 0.64 |
| Full-fat milk | 97.1 | 58.8 | 142.4 | 71.5 | 44.6 | 104.5 | -26.4 | 0.44 |  | 79.4 | 50.0 | 114.1 | 67.9 | 45.1 | 125.1 | -14.5 | 0.50 |  | 35.4 | 10.1 | 65.9 | 45.1 | 6.8 | 86.9 | 27.4 | 0.62 |
| Low-fat milk | 0.1 | 0.0 | 0.3 | 0.0 | 0.0 | 0.0 | -100.0 | 0.31 |  | 0.1 | 0.0 | 0.5 | 0.0 | 0.0 | 0.0 | -100.0 | 0.34 |  | 0.1 | 0.0 | 0.4 | 0.0 | 0.0 | 0.0 | -100.0 | 0.12 |
| Yoghurt | 11.4 | 0.0 | 24.0 | 14.4 | 3.8 | 26.3 | 26.6 | 0.66 |  | 10.0 | 2.5 | 19.1 | 10.5 | 3.5 | 25.0 | 5.6 | 0.49 |  | 8.1 | 0.0 | 16.8 | 6.7 | 2.2 | 21.0 | -16.6 | 0.54 |
| Cheese | 2.1 | 1.0 | 3.9 | 1.9 | 0.9 | 3.4 | -10.1 | 0.34 |  | 1.9 | 0.8 | 3.7 | 2.0 | 1.1 | 4.6 | 2.6 | 0.34 |  | 1.6 | 0.7 | 3.2 | 1.7 | 0.9 | 4.6 | 9.0 | 0.41 |
| Fat and oil | 5.7 | 4.4 | 7.0 | 6.3 | 5.2 | 8.3 | 11.2 | 0.13 |  | 5.7 | 4.7 | 6.9 | 6.0 | 4.8 | 7.7 | 6.0 | 0.18 |  | 5.9 | 4.4 | 7.2 | 6.5 | 5.1 | 8.4 | 10.2 | 0.22 |
| Sugar and confectionaries | 29.0 | 18.0 | 40.2 | 38.0 | 26.2 | 53.3 | 31.0 | 0.34 |  | 22.3 | 12.9 | 34.7 | 40.1 | 25.1 | 57.7 | 79.9 | 0.31 |  | 15.3 | 8.9 | 26.4 | 32.9 | 20.0 | 44.5 | 115.2 | 0.54 |
| Sugar | 4.7 | 2.9 | 6.1 | 2.0 | 1.3 | 3.1 | -56.3 | 0.08 |  | 4.0 | 2.8 | 5.7 | 1.8 | 1.2 | 2.6 | -56.0 | 0.12 |  | 3.9 | 2.5 | 5.3 | 1.3 | 0.8 | 2.4 | -67.0 | 0.15 |
| Confectionaries | 24.7 | 14.1 | 34.2 | 35.8 | 23.9 | 51.4 | 44.9 | 0.34 |  | 18.5 | 9.1 | 29.5 | 37.8 | 22.9 | 54.5 | 104.1 | 0.30 |  | 11.4 | 4.9 | 19.9 | 31.1 | 16.6 | 41.4 | 173.7 | 0.48 |
| Beverages | 290 | 203 | 406 | 334 | 238 | 430 | 15.1 | 0.33 |  | 298 | 206 | 384 | 328 | 246 | 429 | 10.3 | 0.25 |  | 312 | 241 | 407 | 432 | 290 | 539 | 38.2 | 0.32 |
| Water | 38.1 | 7.0 | 96.5 | 63.6 | 7.2 | 180.0 | 67.1 | 0.31 |  | 34.9 | 5.6 | 90.9 | 82.2 | 9.6 | 208.9 | 135.6 | 0.29 |  | 39.8 | 14.8 | 97.4 | 94.9 | 15.8 | 195.5 | 138.2 | 0.26 |
| Tea | 166 | 77 | 252 | 196 | 65 | 277 | 17.8 | 0.42 |  | 139 | 61 | 244 | 157 | 47 | 242 | 12.2 | 0.43 |  | 150 | 92 | 235 | 180 | 77 | 263 | 19.6 | 0.40 |
| Fruit and vegetable juice | 0.4 | 0.0 | 13.2 | 7.9 | 0.0 | 22.6 | 1702.3 | 0.32 |  | 0.2 | 0.0 | 16.0 | 7.1 | 0.0 | 24.2 | 3850.0 | 0.39 |  | 6.9 | 0.0 | 19.5 | 20.8 | 5.8 | 51.3 | 199.4 | 0.44 |
| Sugar-sweetened beverages | 33.9 | 12.6 | 71.7 | 37.2 | 16.0 | 59.8 | 9.8 | 0.42 |  | 44.8 | 18.1 | 89.8 | 42.1 | 16.9 | 73.0 | -5.9 | 0.49 |  | 51.2 | 20.8 | 100.0 | 60.2 | 28.2 | 137.1 | 17.5 | 0.42 |
| Seasonings | 54.5 | 43.4 | 72.5 | 9.9 | 7.9 | 12.8 | -81.8 | 0.26 |  | 48.1 | 35.9 | 64.1 | 9.1 | 7.0 | 11.9 | -81.1 | 0.05 |  | 36.3 | 28.2 | 55.7 | 8.7 | 6.9 | 11.1 | -76.0 | 0.10 |
|  |  |  |  |  |  |  |  |  |  |  |  |  |  |  |  |  |  |  |  |  |  |  |  |  |  |  |
| Median |  |  |  |  |  |  |  | 0.34 |  |  |  |  |  |  |  |  | 0.31 |  |  |  |  |  |  |  |  | 0.40 |

P25, 25th percentile; P75, 75th percentile.

^*^ Percentage differences: (BDHQ15y – 8-day DR)/8-day DR × 100 (%).

**Table ST7.** Median estimates of energy-adjusted food group intakes using the density method from the 8-day weighed dietary record (DR) and the brief-type diet history questionnaire for Japanese adolescents and children (BDHQ15y) and their correlations in 412 Japanese girls aged 6–17 years: percentage differences in median intakes and Spearman’s rank correlations coefficients (CCs), according to the age group.

| Food group (g/1000 kcal)) | 6–9 years (*n* 144) | | | | | | | |  | 10–14 years (*n* 162) | | | | | | | |  | 15–17 years (*n* 106) | | | | | | | |
| --- | --- | --- | --- | --- | --- | --- | --- | --- | --- | --- | --- | --- | --- | --- | --- | --- | --- | --- | --- | --- | --- | --- | --- | --- | --- | --- |
|  | 8-day DR | | | BDHQ15y | | | Median difference (%)^*^ | CC |  | 8-day DR | | | BDHQ15y | | | Median difference (%)^*^ | CC |  | 8-day DR | | | BDHQ15y | | | Median difference (%)^*^ | CC |
|  | Median | P25 | P75 | Median | P25 | P75 |  |  |  | Median | P25 | P75 | Median | P25 | P75 |  |  |  | Median | P25 | P75 | Median | P25 | P75 |  |  |
| Grains | 209 | 186 | 234 | 222 | 181 | 249 | 6.5 | 0.17 |  | 212 | 189 | 237 | 213 | 180 | 249 | 0.7 | 0.30 |  | 216 | 192 | 240 | 224 | 185 | 258 | 3.4 | 0.27 |
| Rice | 133 | 109 | 160 | 167 | 123 | 200 | 26.0 | 0.19 |  | 139 | 119 | 164 | 164 | 121 | 206 | 18.2 | 0.31 |  | 147 | 124 | 178 | 181 | 128 | 218 | 23.3 | 0.37 |
| Bread | 23.0 | 13.6 | 32.5 | 16.0 | 11.1 | 26.2 | -30.6 | 0.46 |  | 23.9 | 16.4 | 33.6 | 20.6 | 10.1 | 30.3 | -13.7 | 0.27 |  | 20.5 | 9.5 | 29.0 | 14.4 | 6.8 | 30.1 | -30.0 | 0.33 |
| Noodles | 38.5 | 20.3 | 55.5 | 29.4 | 20.8 | 41.5 | -23.7 | 0.30 |  | 32.5 | 17.7 | 46.2 | 26.7 | 18.0 | 36.2 | -17.9 | 0.15 |  | 32.8 | 16.5 | 52.9 | 26.1 | 17.4 | 33.9 | -20.2 | 0.31 |
| Potatoes | 25.0 | 17.9 | 33.1 | 16.9 | 10.3 | 21.5 | -32.7 | 0.17 |  | 22.1 | 16.7 | 29.8 | 16.8 | 10.5 | 27.0 | -24.2 | 0.12 |  | 20.9 | 13.9 | 30.3 | 15.4 | 8.0 | 23.5 | -26.0 | 0.21 |
| Pulses | 18.4 | 10.9 | 27.4 | 21.2 | 15.0 | 32.1 | 15.7 | 0.46 |  | 17.2 | 11.2 | 25.0 | 22.9 | 13.6 | 33.0 | 33.0 | 0.18 |  | 16.2 | 9.3 | 25.5 | 25.9 | 11.7 | 36.7 | 60.4 | 0.33 |
| Total vegetables | 112.4 | 87.0 | 137.8 | 100.1 | 75.3 | 136.5 | -11.0 | 0.35 |  | 110.0 | 88.1 | 138.1 | 107.1 | 82.5 | 148.5 | -2.7 | 0.22 |  | 108.4 | 81.0 | 135.9 | 107.8 | 82.9 | 150.4 | -0.6 | 0.35 |
| Green and yellow vegetables | 33.8 | 25.2 | 45.9 | 33.9 | 24.3 | 50.3 | 0.4 | 0.29 |  | 34.6 | 22.1 | 44.7 | 37.2 | 24.4 | 52.2 | 7.2 | 0.18 |  | 31.7 | 22.3 | 45.7 | 36.1 | 24.6 | 56.4 | 13.9 | 0.40 |
| Other vegetables | 65.3 | 47.5 | 81.1 | 50.3 | 38.9 | 73.3 | -23.0 | 0.29 |  | 66.5 | 55.1 | 82.6 | 59.3 | 41.2 | 80.0 | -10.8 | 0.24 |  | 62.8 | 44.2 | 79.6 | 58.8 | 45.7 | 83.3 | -6.4 | 0.24 |
| Pickled vegetables | 0.5 | 0.1 | 1.7 | 0.7 | 0.0 | 3.0 | 34.0 | 0.20 |  | 0.7 | 0.0 | 2.0 | 1.2 | 0.0 | 3.4 | 80.0 | 0.24 |  | 1.5 | 0.1 | 3.8 | 1.5 | 0.0 | 4.4 | 0.7 | 0.19 |
| Mushrooms | 4.6 | 2.3 | 6.7 | 5.0 | 3.2 | 8.1 | 7.8 | 0.43 |  | 4.1 | 2.7 | 6.5 | 4.8 | 2.1 | 8.2 | 18.1 | 0.41 |  | 4.4 | 2.3 | 7.4 | 5.1 | 2.0 | 9.4 | 15.5 | 0.42 |
| Seaweeds | 3.8 | 2.2 | 6.8 | 4.3 | 2.1 | 7.4 | 12.5 | 0.24 |  | 3.6 | 1.6 | 6.7 | 3.0 | 1.7 | 6.5 | -17.5 | 0.08 |  | 3.3 | 1.1 | 7.0 | 4.0 | 2.0 | 6.6 | 21.5 | 0.28 |
| Fruits | 29.2 | 14.0 | 49.5 | 37.0 | 19.1 | 60.2 | 26.5 | 0.50 |  | 23.6 | 10.9 | 42.5 | 26.5 | 14.2 | 50.5 | 12.2 | 0.57 |  | 21.6 | 6.7 | 38.9 | 32.1 | 10.2 | 53.9 | 48.5 | 0.56 |
| Fish and shellfish | 20.9 | 12.9 | 27.7 | 29.0 | 20.3 | 38.1 | 38.8 | 0.35 |  | 19.2 | 12.7 | 29.7 | 28.0 | 19.9 | 38.7 | 45.6 | 0.19 |  | 20.1 | 12.0 | 27.3 | 28.1 | 20.4 | 42.4 | 39.4 | 0.27 |
| Meat | 45.0 | 35.6 | 55.1 | 34.5 | 28.0 | 46.2 | -23.4 | 0.27 |  | 49.8 | 40.4 | 62.5 | 37.4 | 29.2 | 45.4 | -24.9 | 0.16 |  | 56.5 | 47.8 | 69.7 | 44.9 | 32.9 | 54.3 | -20.5 | 0.24 |
| Eggs | 18.1 | 13.5 | 26.5 | 14.9 | 10.6 | 22.7 | -17.7 | 0.12 |  | 21.3 | 16.0 | 28.4 | 18.6 | 12.1 | 28.2 | -12.6 | 0.39 |  | 26.1 | 19.8 | 34.3 | 25.4 | 16.4 | 33.1 | -2.5 | 0.42 |
| Dairy products | 115.1 | 86.0 | 156.4 | 99.0 | 73.4 | 154.2 | -14.0 | 0.44 |  | 93.1 | 60.7 | 132.3 | 92.7 | 59.0 | 130.3 | -0.4 | 0.54 |  | 43.1 | 24.2 | 76.1 | 41.2 | 11.6 | 67.9 | -4.5 | 0.67 |
| Full-fat milk | 97.7 | 63.3 | 130.1 | 77.7 | 56.0 | 119.0 | -20.4 | 0.36 |  | 66.5 | 42.8 | 101.5 | 66.2 | 34.4 | 101.7 | -0.5 | 0.55 |  | 17.1 | 4.5 | 45.9 | 10.0 | 0.0 | 42.8 | -41.6 | 0.72 |
| Low-fat milk | 0.2 | 0.0 | 0.3 | 0.0 | 0.0 | 0.0 | -100.0 | 0.22 |  | 0.1 | 0.0 | 0.7 | 0.0 | 0.0 | 0.0 | -100.0 | 0.13 |  | 0.0 | 0.0 | 0.3 | 0.0 | 0.0 | 0.0 | -100.0 | 0.16 |
| Yoghurt | 11.3 | 4.2 | 24.4 | 8.2 | 3.9 | 22.5 | -27.9 | 0.59 |  | 8.7 | 0.0 | 21.5 | 8.0 | 3.1 | 21.1 | -8.5 | 0.37 |  | 12.5 | 1.1 | 28.0 | 7.2 | 3.0 | 28.9 | -42.5 | 0.52 |
| Cheese | 2.3 | 1.0 | 4.0 | 2.2 | 1.1 | 4.7 | -7.3 | 0.26 |  | 2.4 | 0.8 | 4.1 | 1.6 | 0.9 | 4.3 | -33.3 | 0.23 |  | 1.9 | 0.4 | 4.5 | 1.6 | 0.7 | 5.0 | -16.7 | 0.48 |
| Fat and oil | 5.7 | 4.5 | 7.2 | 6.9 | 5.3 | 8.5 | 20.7 | 0.24 |  | 6.3 | 4.8 | 7.9 | 7.2 | 5.6 | 8.8 | 14.0 | 0.18 |  | 6.4 | 4.9 | 8.2 | 7.2 | 5.5 | 9.6 | 13.1 | 0.11 |
| Sugar and confectionaries | 31.2 | 20.6 | 40.6 | 8.2 | 6.8 | 10.1 | 38.4 | 0.38 |  | 26.0 | 15.9 | 38.9 | 40.3 | 27.7 | 57.8 | 55.0 | 0.35 |  | 26.5 | 18.7 | 34.9 | 40.9 | 25.5 | 62.7 | 54.3 | 0.30 |
| Sugar | 4.7 | 2.9 | 6.2 | 2.3 | 1.4 | 3.5 | -52.3 | 0.09 |  | 4.7 | 3.3 | 6.6 | 2.0 | 1.5 | 3.1 | -56.9 | 0.28 |  | 4.4 | 3.0 | 6.6 | 1.7 | 0.8 | 2.6 | -62.6 | 0.02 |
| Confectionaries | 26.5 | 15.8 | 34.9 | 39.6 | 26.4 | 54.8 | 49.1 | 0.36 |  | 20.5 | 12.0 | 32.7 | 37.4 | 24.1 | 53.5 | 82.6 | 0.34 |  | 19.9 | 13.2 | 30.9 | 39.1 | 23.1 | 61.2 | 96.7 | 0.33 |
| Beverages | 290 | 195 | 400 | 350 | 269 | 459 | 20.8 | 0.24 |  | 282 | 196 | 380 | 356 | 275 | 451 | 25.8 | 0.13 |  | 336 | 246 | 413 | 411 | 280 | 527 | 22.4 | 0.12 |
| Water | 37.3 | 9.3 | 100.6 | 58.5 | 6.3 | 195.9 | 56.9 | 0.24 |  | 29.2 | 9.0 | 97.3 | 61.9 | 5.8 | 203.7 | 111.9 | 0.38 |  | 47.5 | 15.0 | 107.8 | 60.3 | 4.9 | 258.3 | 27.0 | 0.16 |
| Tea | 160 | 77 | 246 | 215 | 79 | 284 | 34.6 | 0.33 |  | 162 | 81 | 251 | 203 | 104 | 272 | 25.1 | 0.35 |  | 195 | 123 | 288 | 238 | 101 | 333 | 22.1 | 0.43 |
| Fruit and vegetable juice | 0.1 | 0.0 | 17.0 | 9.2 | 0.0 | 26.0 | 7558.3 | 0.41 |  | 0.9 | 0.0 | 16.2 | 8.5 | 0.0 | 28.6 | 821.7 | 0.51 |  | 0.3 | 0.0 | 14.8 | 10.1 | 0.0 | 30.8 | 2879.4 | 0.36 |
| Sugar-sweetened beverages | 29.3 | 5.2 | 65.0 | 28.3 | 10.4 | 55.5 | -3.4 | 0.52 |  | 27.6 | 2.6 | 57.6 | 31.1 | 13.1 | 50.8 | 12.7 | 0.37 |  | 31.8 | 9.6 | 88.5 | 23.5 | 7.5 | 61.7 | -26.1 | 0.41 |
| Seasonings | 59.1 | 44.3 | 79.3 | 10.1 | 7.7 | 12.3 | -82.8 | 0.11 |  | 50.9 | 38.5 | 64.8 | 10.5 | 8.3 | 13.3 | -79.4 | 0.06 |  | 44.3 | 32.2 | 58.7 | 9.8 | 7.0 | 11.8 | -77.9 | 0.14 |
|  |  |  |  |  |  |  |  |  |  |  |  |  |  |  |  |  |  |  |  |  |  |  |  |  |  |  |
| Median |  |  |  |  |  |  |  | 0.29 |  |  |  |  |  |  |  |  | 0.27 |  |  |  |  |  |  |  |  | 0.33 |

P25, 25th percentile; P75, 75th percentile.

^*^ Percentage differences: (BDHQ15y – 8-day DR)/8-day DR × 100 (%).
